# Nonlinear behavior of fixed-end RC beams with circular openings based on experimental and finite element analysis

**DOI:** 10.1038/s41598-026-56645-9

**Published:** 2026-06-11

**Authors:** Huseyin Hilmi Aslanbay, Fatih Altun

**Affiliations:** Engineering Faculty, Civil Engineering Department, Erciyes University, Kayseri, Turkey

**Keywords:** RC beams with openings, Fixed support, Behavior of flexural, Ductility, ABAQUS, Engineering, Materials science

## Abstract

Reinforced concrete (RC) beams often require service openings due to architectural and utility demands. However, such openings lead to stress concentrations caused by the reduction in the concrete cross-section and the absence of transverse reinforcement, which can adversely affect the structural performance of beams and lead to premature failures. Despite extensive research on RC beams with openings, most studies have been conducted under simply supported conditions, which do not accurately represent real structural behavior. This study aims to investigate the structural behavior of RC beams with circular mid-span openings under fixed-end (encastre) boundary conditions through both experimental testing and numerical analysis. Openings were introduced in the bending region at 20%, 40%, and 60% of the beam height. Full-scale beam specimens were tested under four-point bending, and detailed nonlinear finite element models were developed using ABAQUS software. The results show that openings exceeding 40% of the beam height significantly reduce ductility and lead to sudden load drops. Additionally, when openings exceed 20%, the remaining concrete section around the opening plays a critical role in load transfer and bond performance. The numerical results exhibit strong agreement with the experimental findings, demonstrating that the proposed modeling approach can reliably capture the structural behavior of beams with openings. The findings provide important insights into the realistic behavior of fixed-end RC beams and contribute to the safe and efficient design of structural members with service openings.

**Supplementary Information:**

The online version contains supplementary material available at 10.1038/s41598-026-56645-9.

## Introduction

In modern structural engineering applications, it is often necessary to introduce openings in reinforced concrete (RC) beams to accommodate architectural and service requirements such as electrical, ventilation, plumbing, and HVAC systems. However, these openings interrupt the continuity of the load-carrying mechanism and may significantly affect the structural performance of beams if not properly considered during design. Uncontrolled or improperly positioned web openings can lead to reductions in load-carrying capacity, stiffness, ductility, and energy dissipation, ultimately compromising the safety and serviceability of the structure.

A large body of research has demonstrated that the mechanical response of RC beams is highly sensitive to the geometric characteristics of web openings. In particular, the size, shape, and location of openings play a critical role in governing stress distribution, crack initiation, and failure mechanisms. Openings located within shear-critical regions have been shown to cause earlier crack formation and more brittle failure compared to those positioned in flexural regions^1–3^. In addition, circular openings generally exhibit more favorable behavior than rectangular ones due to smoother stress flow around the opening boundaries^4–6^. Beyond geometric considerations, the presence of openings fundamentally alters the internal force transfer mechanism of RC beams. Previous studies have shown that openings introduce stress concentrations and disrupt load paths, resulting in reduced stiffness, strength, and ductility^7–13^. This disruption also leads to more complex deformation patterns and modified crack propagation paths, with cracks forming earlier and propagating more rapidly compared to solid beams^14–16^. As a result, beams with openings exhibit a noticeable reduction in structural integrity and a shift in governing failure modes^17^.

Experimental investigations further indicate that increasing opening size leads to significant reductions in ultimate load capacity, cracking load, displacement ductility, and energy absorption capacity^3,18–20^. These effects become more pronounced when openings are located near critical regions such as supports or beam–column joints, where shear forces dominate. In such cases, the load transfer mechanism is severely disturbed, leading to substantial decreases in stiffness and load-carrying capacity, as well as changes in failure characteristics^5,21–24^. The influence of openings is not limited to flexural behavior but also extends to torsional response and overall structural deformation. Studies on beams with varying cross-sectional configurations have shown that the height and position of openings significantly affect torsional stiffness and rotational capacity^25,26^. Similarly, openings located near compression zones or within shear spans tend to result in more severe strength degradation compared to those in pure flexural regions^2,3,27^.

Recent studies have also highlighted that web openings affect both global and local structural responses by increasing deflection, altering crack propagation patterns, and modifying failure modes depending on their geometry and location^28–30^. Furthermore, it has been suggested that the opening size should be limited relative to beam depth to prevent excessive reductions in structural performance, particularly in shear-dominated regions^3,31,32^. Numerical studies have confirmed that openings positioned near supports can lead to drastic reductions in stiffness and load-carrying capacity^33^. In addition, experimental investigations on full-scale beams have demonstrated that opening size significantly influences flexural behavior and deformation capacity, while increasing opening depth leads to further reductions in structural performance^30^. Complementary numerical analyses have also shown that the presence of web openings significantly affects shear response and stress distribution, particularly in beams with square openings, highlighting the sensitivity of structural behavior to opening geometry^34^.

Despite the extensive research on RC beams with web openings, several limitations remain in the existing literature. Many previous studies have been conducted under simply supported boundary conditions or on scaled specimens, which may not accurately represent the actual behavior of structural members in real applications. In addition, a significant portion of the available research focuses on isolated aspects of behavior or specific intervention strategies, rather than providing a comprehensive evaluation of the mechanical response of beams with openings under realistic boundary conditions. These limitations highlight the need for systematic investigations that consider both full-scale configurations and realistic support conditions. In particular, the behavior of RC beams with web openings under fixed-end boundary conditions, where both rotational and translational restraints are present, has not been sufficiently explored.

In this context, the present study aims to address this research gap by investigating the flexural behavior, deformation characteristics, and failure mechanisms of full-scale RC beams with circular openings under fixed-end conditions. The openings are located at the mid-span flexural region and vary in size, corresponding to 20%, 40%, and 60% of the beam depth. An integrated experimental–numerical framework is adopted, including full-scale monotonic loading tests and nonlinear finite element modeling using ABAQUS to simulate and interpret the observed behavior. The novelty of this study lies in the realistic representation of boundary conditions in both experimental and numerical models, enabling a more accurate assessment of how opening size influences structural performance. The findings are expected to provide a clearer understanding of the mechanical behavior of RC beams with web openings and to contribute to the development of safer and more reliable design approaches for structural members with unavoidable service openings.

## Methodology

### Material properties

**Fig. 1:**
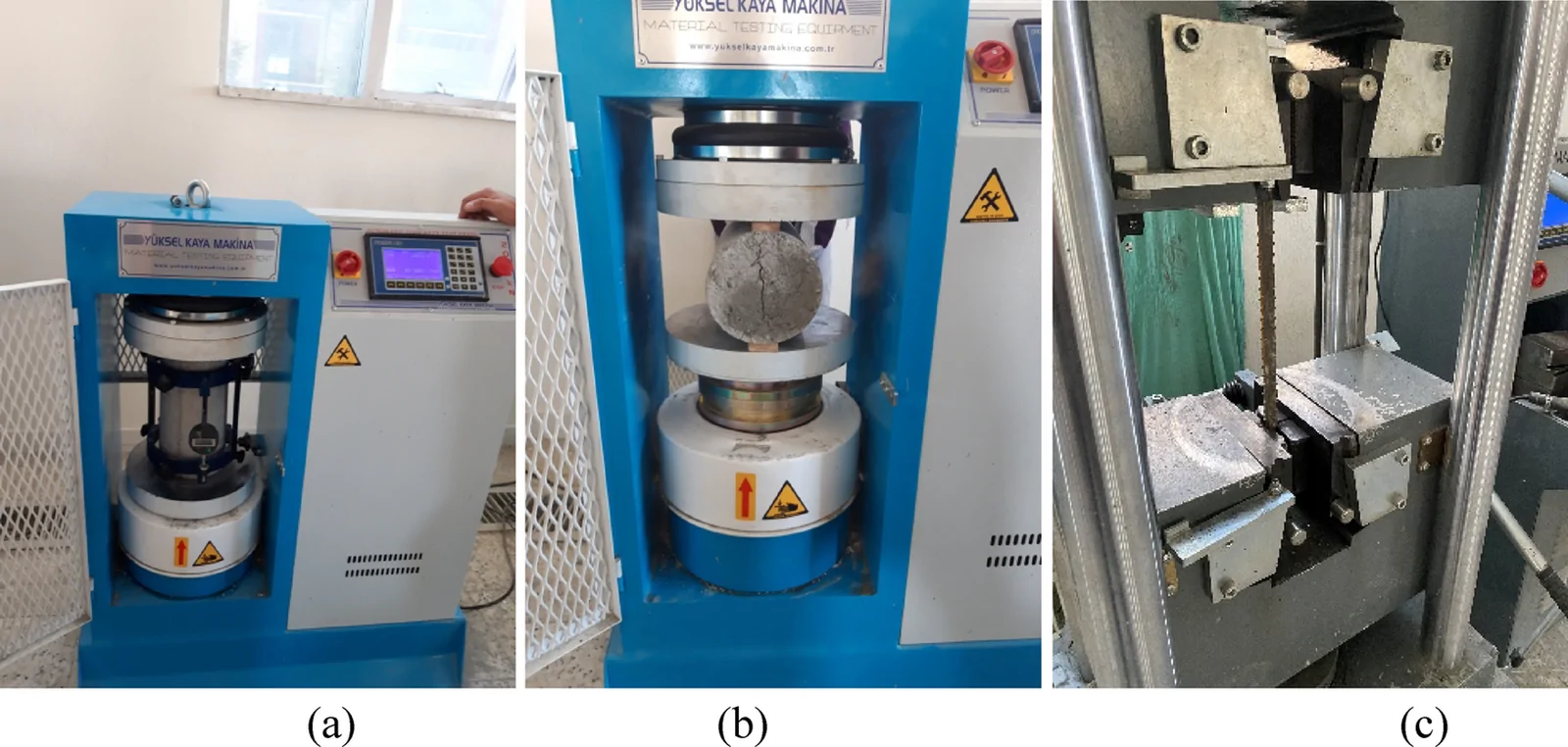
Material tests OPC and Reinforcement samples: (**a**) Concrete compressive tests, (**b**) Concrete splitting tensile tests, (**c**) Reinforcement tensile tests.

Six standard cylindrical specimens with dimensions of 150 mm in diameter and 300 mm in height, produced from Ordinary Portland Cement (OPC) concrete, were tested under uniaxial compression in accordance with ASTM C39 to determine the compressive strength^35^. Splitting tensile strength tests were performed on cylindrical samples following ASTM C496 procedures^36^. In addition, tensile tests were conducted on the reinforcing steel bars to evaluate their yield and ultimate tensile strengths, in accordance with ASTM A370^37^. The obtained mechanical properties were used as input parameters for both the experimental evaluations and the numerical modeling phase. The detailed results of the material tests for concrete and reinforcement are presented in Table 1. Images of material tests are presented in Fig. 1.

**Table 1:** Material mechanical properties OPC and reinforcement.

Sample name	f_c_ (MPa) average	f_t_ (MPa) average	E_c_ (MPa) average	υ, Poisson’s ratio
OPC	29.93	2.06	30,100	0.18
**Diameter (mm)**	**Yield Strength (** $\:{\boldsymbol{\sigma\:}}_{\boldsymbol{s}})$ **(MPa)**	**UltimateTensile Strength (** $\:{\boldsymbol{\sigma\:}}_{\boldsymbol{t}})$ **(MPa)**	**Plastic Strain**	**Elongation at Break** **(%)**
Φ8	520	616	0.1527	16.2
Φ12	440	572	0.1715	20.9
Φ14	432	564	0.1986	21.2

### Experimental method

**Fig. 2:**
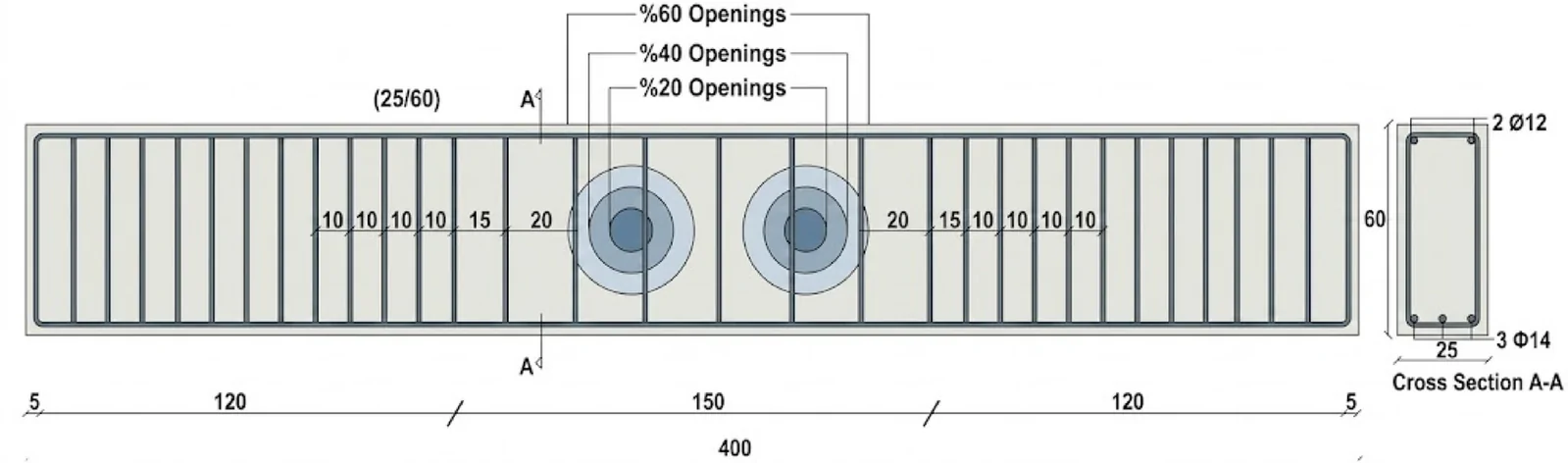
Reinforcement and opening detail of experimental specimens (dimensions in cm).

The reinforced concrete beams had a total length of 4000 mm, a width of 250 mm, and a height of 600 mm. All specimens were manufactured at full scale (1:1) to represent real structural applications. The design and fabrication of the test specimens were carried out in accordance with ACI 318, Eurocode 2, Turkish Standard TS 500–2000, and the Turkish Seismic Code TBEC-2018^38–41^. In the manufacturing process, circular openings were positioned within the bending zone of the beams. Ready-mixed concrete of structural grade and B420C-grade reinforcing steel were used in the specimen production. The reinforcement details of the experimental beams are illustrated in Fig. 2.

In the specimens, the longitudinal reinforcement configuration was identical across all beams, consisting of 3∅14 bars in tension and 2∅12 bars in compression. The transverse reinforcement was arranged as ∅8/100 mm in the shear zone and ∅8/200 mm in the central region. Circular web openings were symmetrically positioned in the bending zone, located 250 mm from the midspan and spaced 500 mm center-to-center. The diameters of the openings corresponded to 20%, 40%, and 60% of the beam height, values selected based on previous research highlighting their influence on stiffness and failure mechanisms^6,24,33,42,43^.

**Fig. 3:**
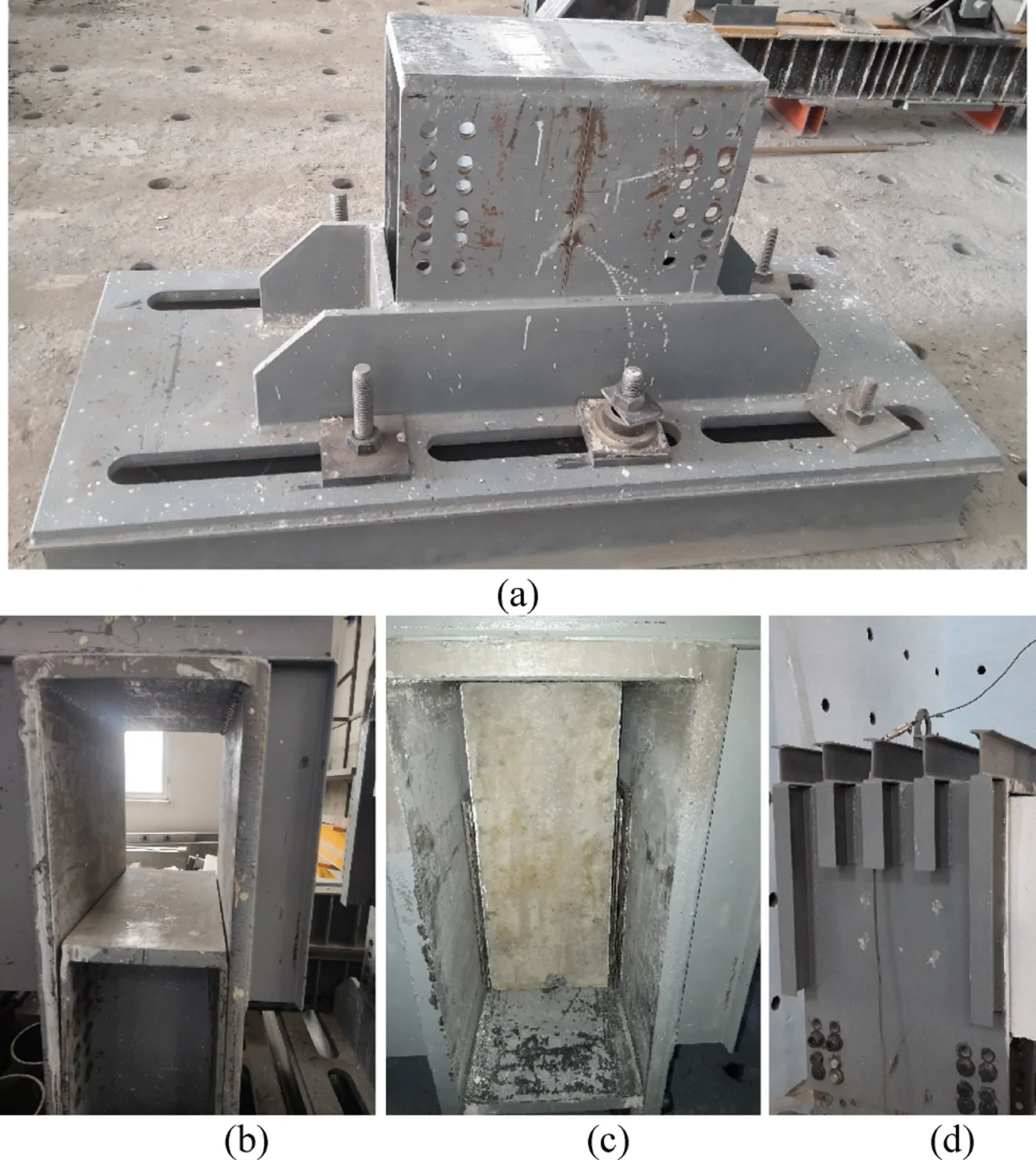
Formation stages of the fixed-end support system; (**a**) anchorage of the lower support component to the gallery opening using threaded rods; (**b**) view of the upper support assembly; (**c**) confinement of the beam specimen within the support system; and (**d**) integration of the upper and lower support heads through bolted connections.

In this study, the tested beams were designed with fixed supports to replicate the actual in-service conditions of structures. The support system was specifically designed to simulate fixed-end boundary conditions by fully restraining both translational and rotational degrees of freedom. As illustrated in Fig. X, long threaded steel rods were passed through the base of the support assembly and extended through the laboratory gallery slab, where they were anchored to the basement level of the laboratory using nuts and bolts, ensuring a rigid connection between the support system and the structural base. After positioning the beam specimen, it was tightly clamped between upper and lower steel plates (support heads). These plates were connected using a dense arrangement of high-strength bolts (approximately 24 bolts per support), providing strong confinement and preventing any relative movement. In addition, steel plates were inserted into the gaps between the beam surfaces and the support elements to ensure full contact and eliminate potential slip or local rotation. This integrated configuration provided a highly rigid connection, effectively enforcing fixed-end boundary conditions. The effectiveness of the support system was also verified through displacement measurements, which showed negligible deflections near the supports during testing (Fig. 3).

**Fig. 4:**
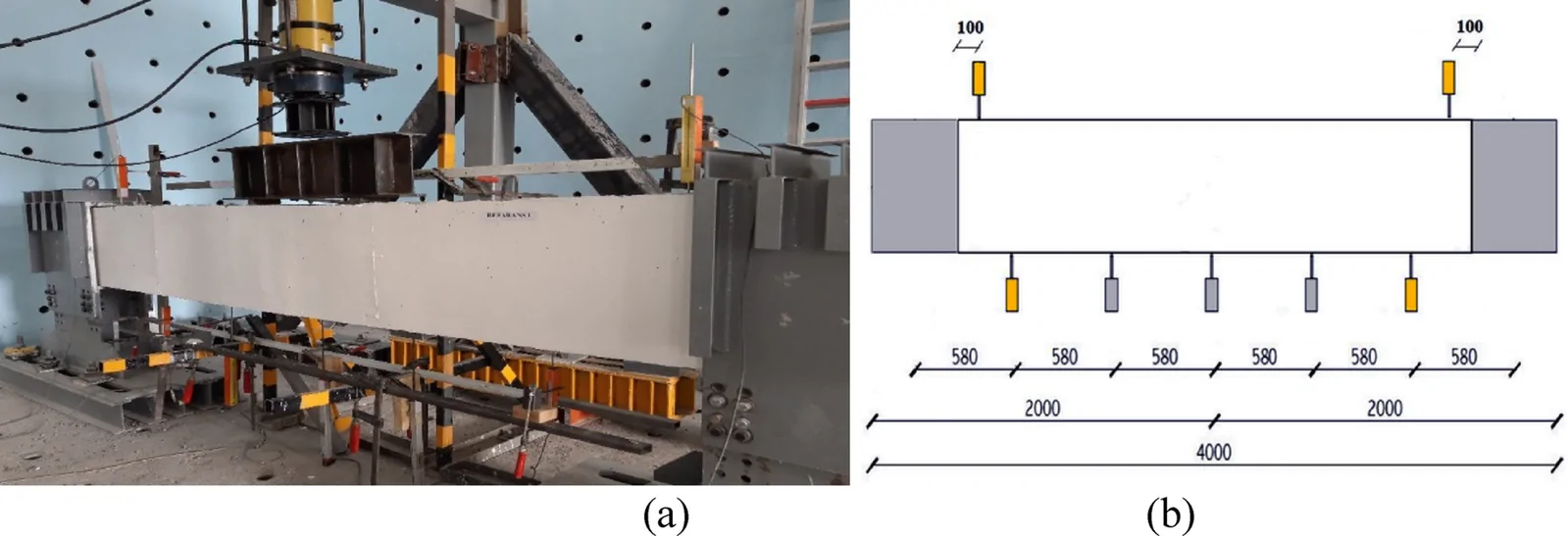
Test setup; (**a**) Loading frame; (**b**) Schematic representation of the LVDTs.

Unlike the simply supported configurations commonly adopted in the literature, this approach prevented rotational movement at the supports, thereby providing realistic boundary conditions and enhancing the accuracy of the structural response representation under loading. Furthermore, this method aimed to more reliably capture the in-situ behavior of beams with openings located at midspan and in the shear regions. The beam specimens were clamped in the slot using steel plates, and the movement and translation of the load-bearing support regions were restricted. The frame system of the experimental setup was supported by steel buttresses for vertical load application and its movement was prevented. The vertical load was applied monotonically from top to bottom with a hydraulic jack connected to the frame system, with four points. The reinforced concrete beam test setup is shown in Fig. 4. The displacements in the specimen under loading were measured with LVDTs. The length of the beam inside the fixed steel support is 350 mm for the fixed support in the experimental setup. The pure span of the beam is 3300 mm. The distance between the loading points is 600 mm. To measure the displacements, 7 LVDTs were mounted. Datalogger was used to transfer the data taken from the LVDTs and the load cell to the computer. The positions of the LVDTs on the experimental setup are shown in Fig. 4. The geometric properties of the test specimens are presented in Table 2.

The specimens evaluated in this research are designated as follows: the solid (opening-free) reference beam is labeled ‘REF’, while the beams containing circular web openings are denoted by the abbreviation ‘BWO’ (Beam Web Opening). The numerical suffix in the BWO labels represents the opening diameter in millimeters. Accordingly, the designations BWO-120, BWO-240, and BWO-360 correspond to specimens with opening diameters of 120 mm, 240 mm, and 360 mm, respectively.

**Table 2:** Nomenclature and dimensional characteristics of beam specimens.

Specimen name	Sizes (mm)	Length (mm)	Percentage of Opening (%)	Diameter of Opening (mm)	Number of Opening
REF	250/600	4000	0	0	0
BWO-120	250/600	4000	20	120	2
BWO-240	250/600	4000	40	240	2
BWO-360	250/600	4000	60	360	2

**Fig. 5:**
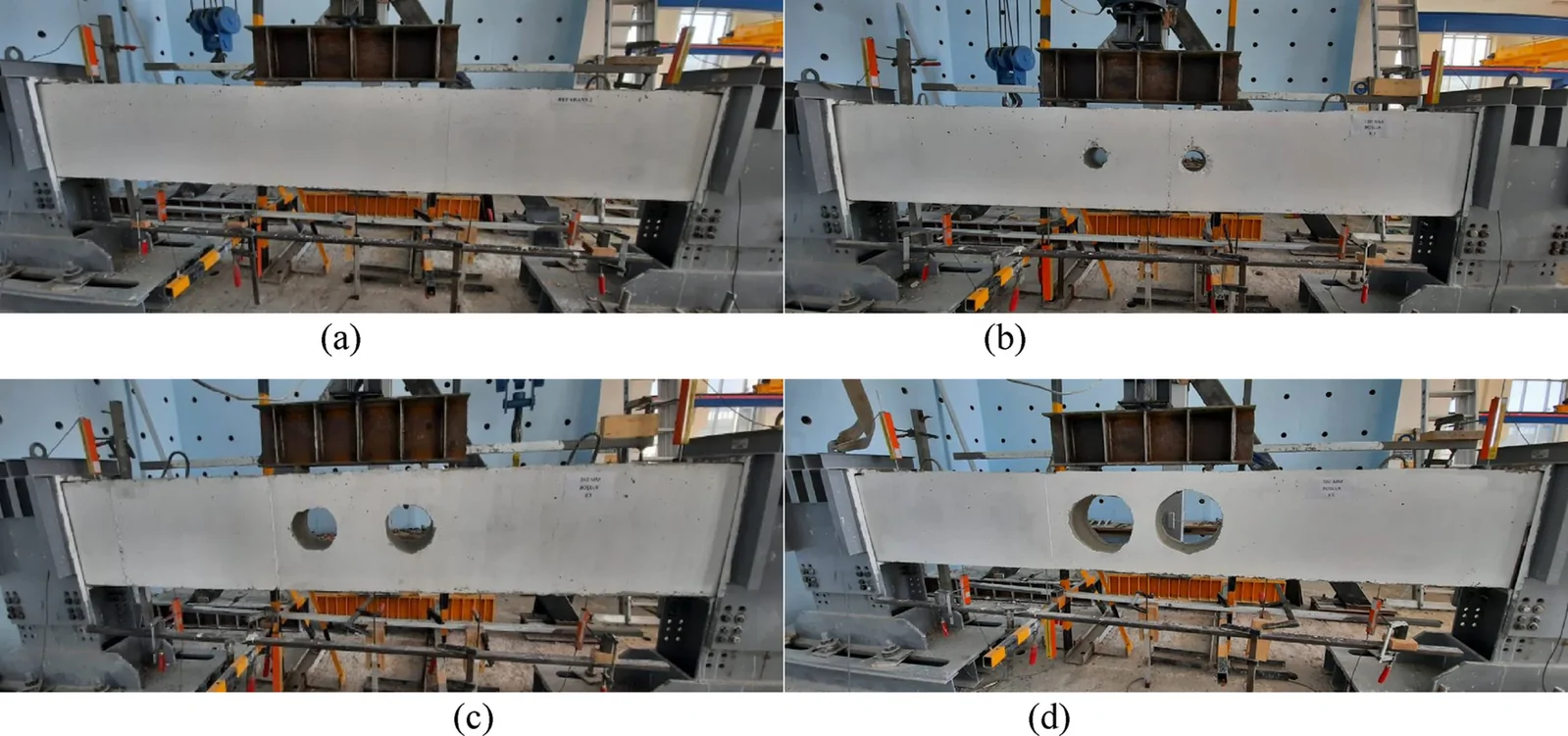
Pre-experiment views of the test specimens; (**a**) specimen “REF”; (**b**) specimen “BWO-120”; (**c**) specimen “BWO-240”; (**d**) specimen “BWO-360”.

Figure 5 presents the pre-experimental views of the test specimens, including fixed support assemblies and LVDT measurement locations, with all beams configured to replicate fixed-end boundary conditions.

A systematic calibration and validation procedure was adopted following a performance-based modeling approach consistent with ASCE/SEI 41 − 23. Material parameters were defined based on experimental data and calibrated through sensitivity analyses, while boundary conditions and loading definitions were refined to match the experimental setup. The numerical model was validated by comparing load–displacement response, peak load, stiffness degradation, and failure modes with experimental results, showing good agreement.

## Numerical method

The numerical modeling and analyses of the tested beams in this study were performed using the finite element software ABAQUS^44^. The finite element software ABAQUS (2018), which is widely utilized for the analysis of reinforced concrete elements^45–47^, was employed to perform the numerical modeling and analyses of the tested beams in this study. To accurately capture the nonlinear behavior under applied loading, the dynamic explicit analysis method was adopted. The SI unit system was utilized throughout the modeling and analysis stages. In the numerical simulations, both elastic and inelastic behaviors were considered to accurately represent the structural response. The concrete elements were modeled using the Concrete Damaged Plasticity (CDP) model, which combines isotropic tensile and compressive plasticity with isotropic damaged elasticity to simulate stiffness degradation and crack propagation under loading^48–53^. The CDP model parameters were derived from laboratory tests conducted on cylindrical specimens (150 mm diameter × 300 mm height) produced from the same OPC concrete mix used in beam fabrication. Uniaxial compression tests were performed in accordance with ASTM C39, while splitting tensile tests were conducted following ASTM C496. The stress–strain curves obtained from these tests for OPC concrete were processed within the framework of the CDP model and implemented as input data in the program.

In this study, all numerical simulations were performed for reinforced concrete members produced with Ordinary Portland Cement (OPC) concrete. The unit weight of OPC concrete was taken as 2.40 g/cm³ in the model definition. The Concrete Damage Plasticity (CDP) model in ABAQUS was employed to simulate the nonlinear behavior of the concrete, accounting for tensile cracking and compressive damage mechanisms. Among the CDP parameters, the eccentricity (ϵ), the ratio of initial biaxial to uniaxial compressive strength ($\:{f}_{b0}/{f}_{c0}$) and the shape factor of the yield surface in the deviatoric plane (K) were adopted from standard recommendations in the ABAQUS documentation, with values of 0.1, 1.16, and 0.667, respectively, as commonly applied in the literature^46,49,54–57^. In contrast, the dilation angle (φ) and viscosity parameter (υ) were determined through parametric analyses aimed at achieving the closest agreement with experimental results. The compressive and splitting tensile test data obtained from laboratory-produced OPC specimens were used to calibrate the input stress–strain relationships for the CDP model, including tensile and compressive strengths, inelastic strain limits, and stiffness degradation characteristics^58^. This calibration ensured that the model accurately represented the mechanical behavior of OPC under both compression and tension. The final CDP parameter set used for OPC concrete in the numerical analysis is presented in Table 3.

**Table 3:** CDP parameters for ABAQUS material definition of OPC^46,49^.

Parameter	Taken value	Description
$\:{f}_{b0}/{f}_{c0}$	1.16	The ratio of initial equibiaxial compressive yield stress to initial uniaxial compressive yield stress. (default)
K	0.6667	$\:{K}_{c}$, the ratio of the second stress invariant on the tensile meridian (default)
$\:\varphi\:$	30	Dilation angle (calibrated)
$\:\upsilon\:$	0.001	Viscosity parameter (calibrated)
$\:\epsilon$	0.1	Eccentricity (default)

### Numerical material modeling

**Fig. 6:**
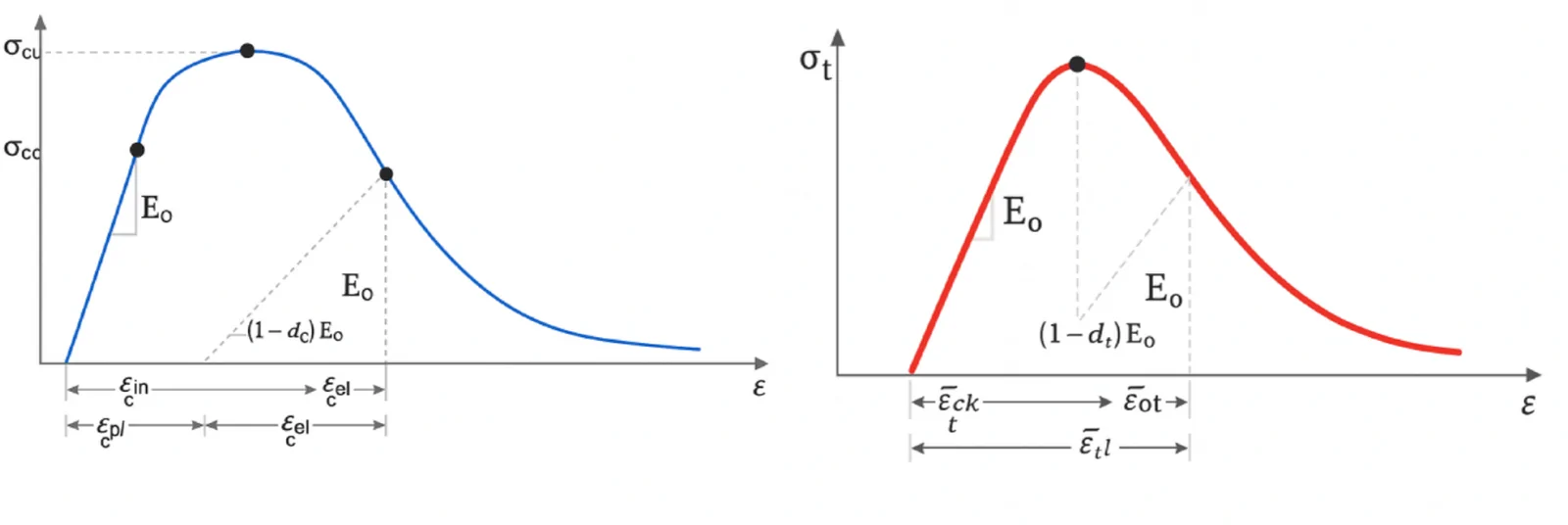
Compression and tensile behavior of concrete; (**a**); Compression behavior (**b**); Tensile behavior^59^.

Within the scope of numerical modeling, the nonlinear behavior of concrete was represented by incorporating both its elastic and inelastic responses into the material definition. The Concrete Damage Plasticity (CDP) model available in ABAQUS was adopted to simulate tensile cracking and compressive crushing mechanisms. The model parameters were calibrated using uniaxial compressive and splitting tensile test results obtained from cylindrical specimens produced during the experimental program. This calibration enabled the CDP model to realistically capture stiffness degradation, damage progression, and crack propagation patterns of concrete under loading. The uniaxial compressive and tensile stress–strain relationships implemented in the CDP model are presented in Fig. 6^59^.

The parameters entered for the compression behavior of concrete in the ABAQUS program are the yield stresses and the inelastic strain values obtained depending on these stresses. In the graph, the inelastic behavior is for after the stress and strain at yield. Inelastic strain values are obtained from Eq. 1^59^. Stress values are calculated according to Eq. 2.

1 $$\varepsilon _c^{in} = {\varepsilon _c} - \frac{{{\sigma _c}}}{{{E_0}}}$$

2 $${\sigma _c} = (1 - {d_c}).{E_0}.({\varepsilon _c} - \varepsilon _c^{pl})$$

The inelastic strain values are calculated and entered into the program and the plastic strain values are calculated by the program. Plastic strains are calculated from Eq. 3. In Eq. 4, $\:{d}_{c}$ represents the damage parameter of concrete under compression loading. The damage parameter refers to the decrease in elastic stiffness depending on the loading after maximum stress. The damage parameter is of great importance in terms of conveying the plastic behavior of concrete in the program as close to reality. The damage parameter is calculated according to Eq. 4. In the equation, the parameter $\:{b}_{c}$ denotes the ratio of plastic strain to inelastic strain. Recent research indicates that, for concrete, this ratio typically falls within the range of 0.5 to 0.7^60–65^. The compression behavior of the concrete along with the properties of the tensile behavior are entered. The tensile behavior of concrete is defined together with its compressive response in the material model. The tensile stress values of concrete $\:({\sigma\:}_{t}$) are obtained from Eq. 5, and the tensile strength is calculated according to Eq. 6. The corresponding inelastic strain values ($\:{\epsilon\:}_{t}^{ck}$) are derived from Eq. 7, while the tensile damage parameter ($\:{d}_{t}$) is determined using Eq. 8. The plastic strain values ($\:{\epsilon\:}_{t}^{pl}$) are calculated within the program. In Eq. 9, the crack width (* w*) is defined, while the mesh size parameter (${I_{eq}}$) represents the characteristic element length^60,62^. The parameter ($\:{b}_{t}$), specified in Eq. 10, is reported in the literature to vary between 0.1 and 0.7^60–65^. Finally, the crack width is evaluated using Eq. 11.

3 $$\varepsilon _c^{pl} = \varepsilon _c^{in} - \frac{{{d_c}}}{{(1 - {d_c})}}\frac{{{\sigma _c}}}{{{E_0}}}$$

4 $${d_c} = 1 - \frac{{{\sigma _c}/{E_0}}}{{{\sigma _c}/{E_0} + \varepsilon _c^{in}(1 - {b_c})}}$$

5 $${\sigma _t} = (1 - {d_t}).{E_0}.({\varepsilon _t} - \varepsilon _t^{pl})$$

6 $$\varepsilon _t^{pl} = \varepsilon _t^{ck} - \frac{{{d_t}}}{{(1 - {d_t})}}\frac{{{\sigma _t}}}{{{E_0}}}$$

7 $$\varepsilon _t^{ck} = {\varepsilon _t} - {\sigma _{t0}}/{E_0}$$

8 $${d_t} = 1 - \frac{{{\sigma _{t0}}/{E_0}}}{{{\sigma _{t0}}/{E_0} + \varepsilon _t^{ck}(1 - {b_t})}}$$

9 $${\varepsilon _t} = {\varepsilon _{t0}} - w/{I_{eq}}$$

10 $${b_t} = \frac{{\varepsilon _t^{pl}}}{{\varepsilon _t^{ck}}}$$

11 $$w = \left[ {\varepsilon _t^{pl} + \frac{{{\sigma _t}.{d_t}}}{{(1 - {d_t}).{E_0}}}} \right].{I_{eq}}$$

**Fig. 7:**
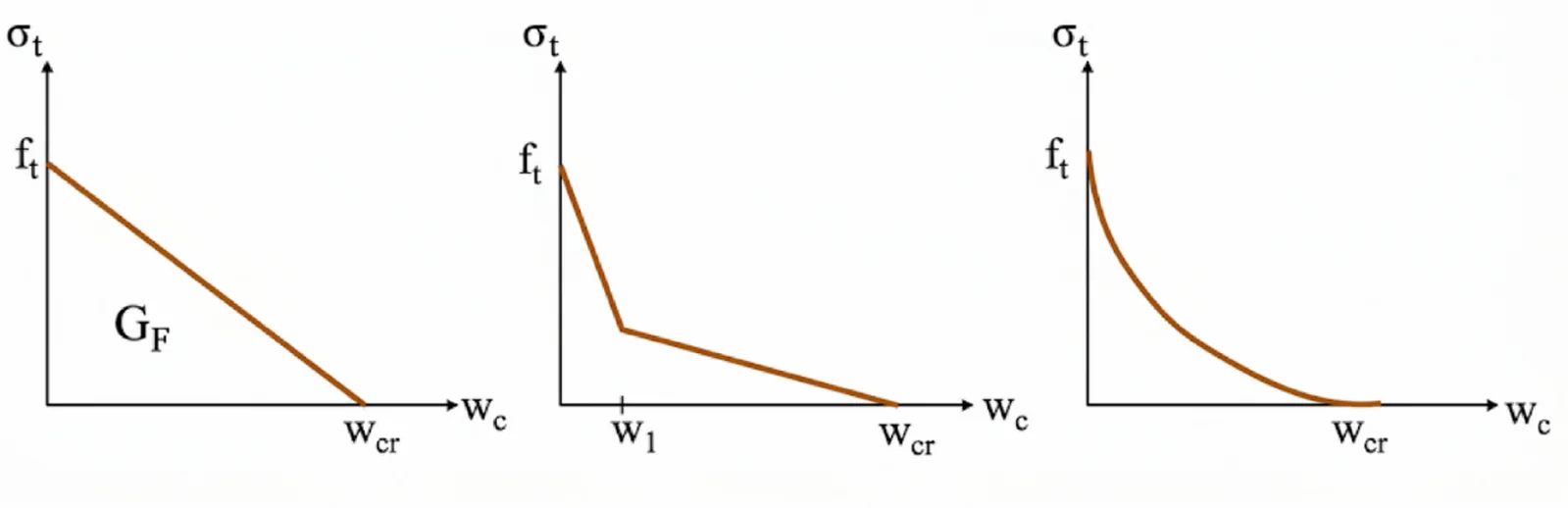
Tensile stiffness models of concrete^66^.

Linear, bilinear, and exponential models of the tensile stiffness of concrete are presented in Fig. 7^66^.

**Fig. 8:**
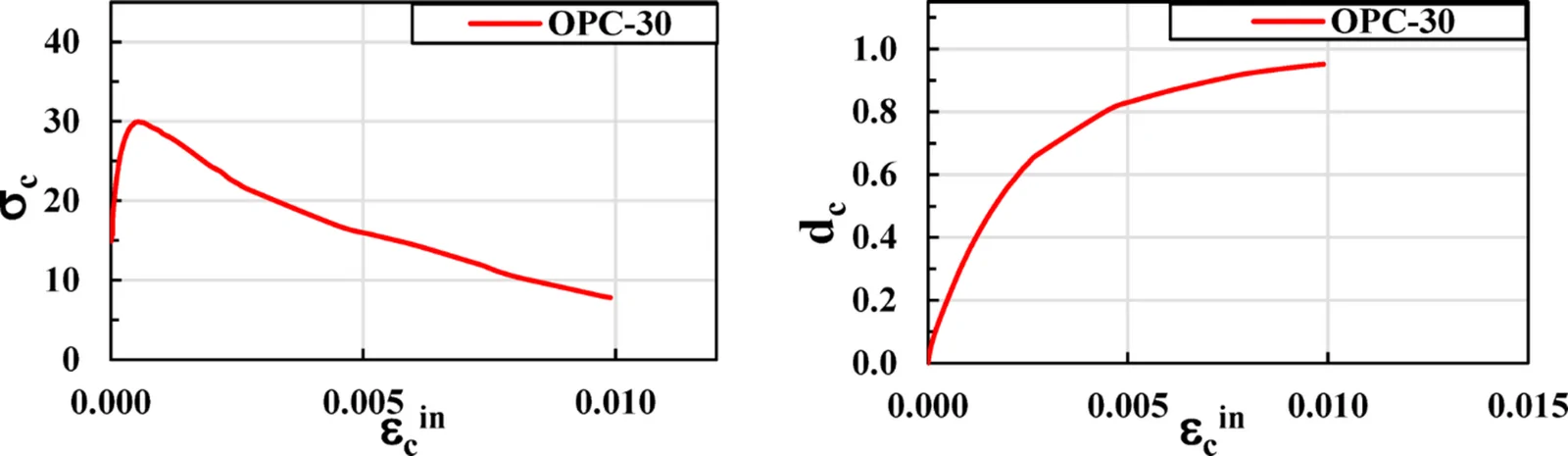
Concrete compression and damage behavior graphs of specimen.

**Fig. 9:**
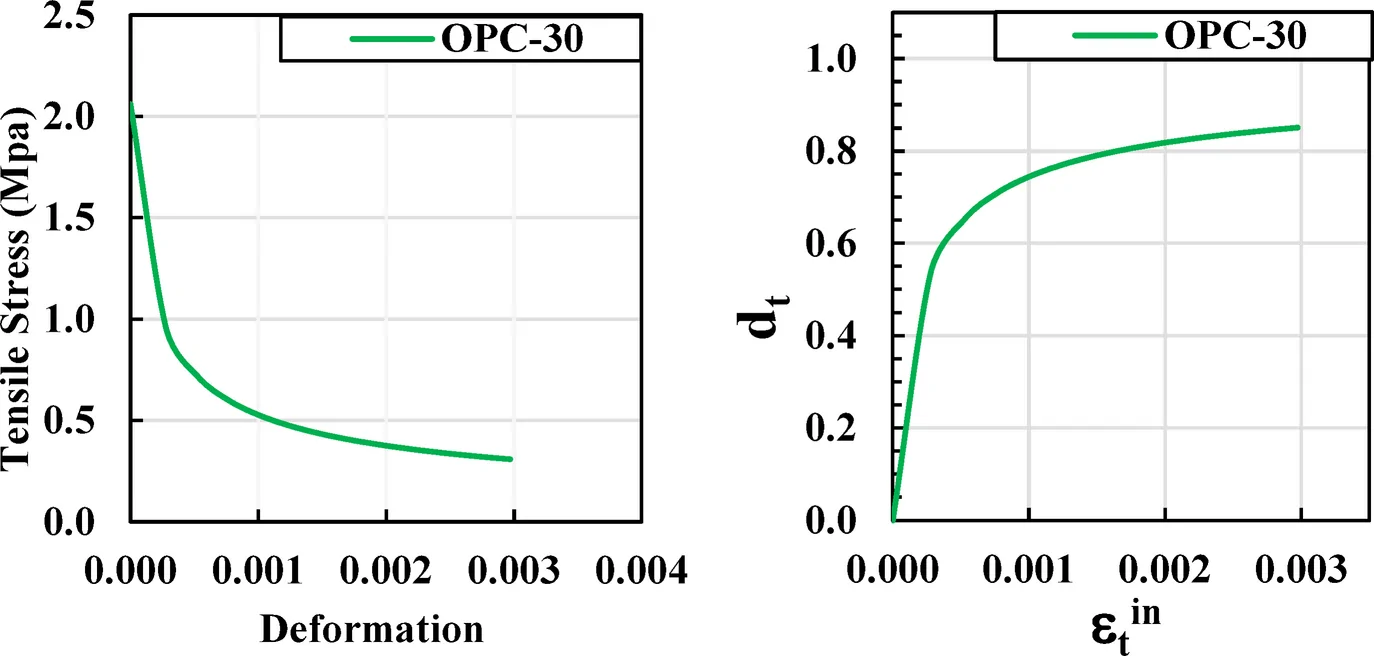
Concrete tensile and damage behavior graphs of specimen.

**Fig. 10:**
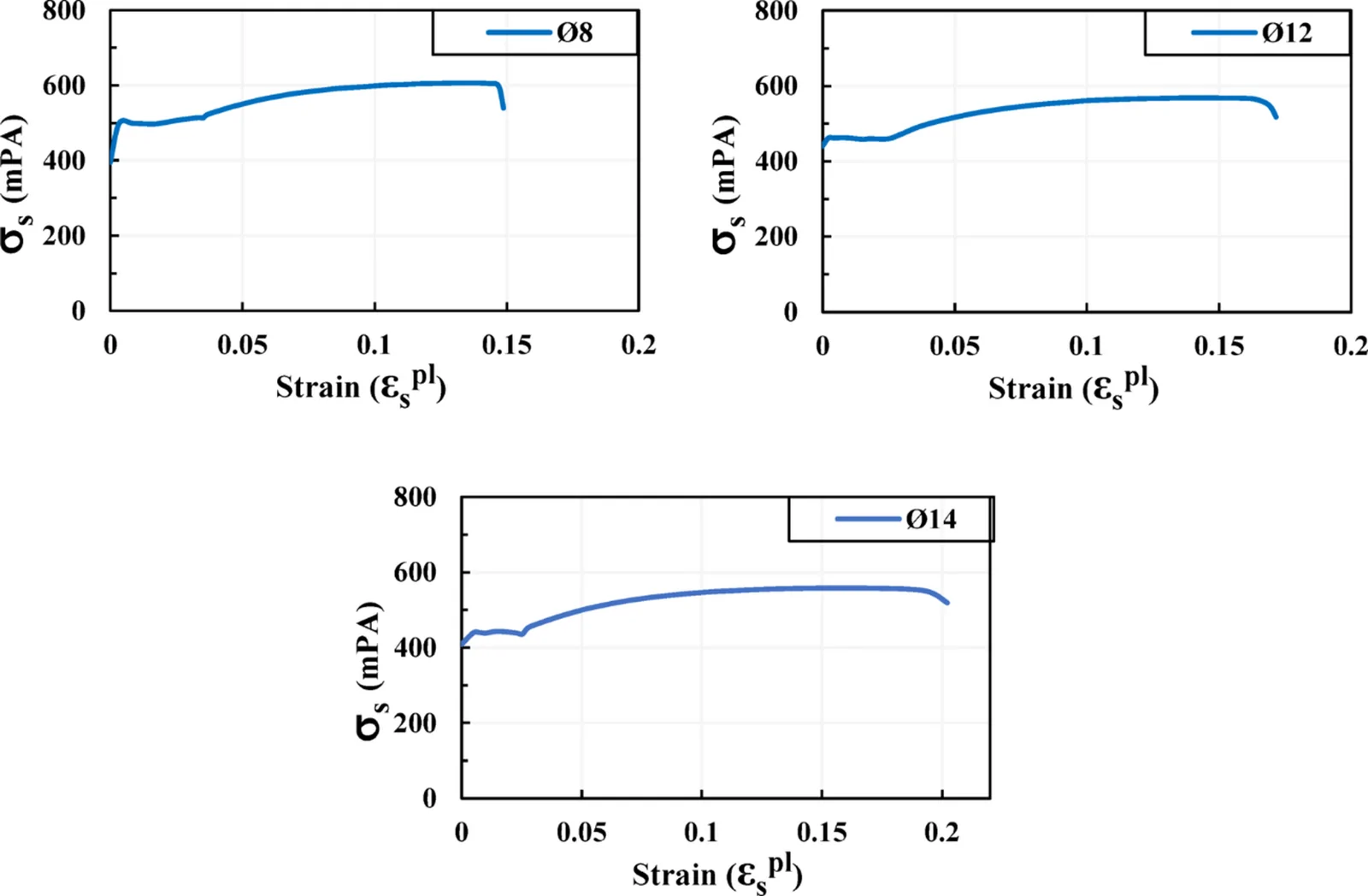
Plastic strain curves of rebar with different diameters.

The properties of the concrete material were created by incorporating all relevant parameters related to the compressive and tensile behavior of the concrete in accordance with the Concrete Damaged Plasticity (CDP) model. The compression and damage behavior of concrete, obtained based on the results of cylinder tests conducted during concrete casting and the governing equations, are presented in Fig. 8. The tensile and damage behavior of concrete are shown in Fig. 9. Additionally, uniaxial tensile tests were performed on the reinforcement bars used in the specimens. The stress–strain data obtained from these tests were converted into stress–plastic strain relationships, considering the bar diameters, and incorporated into the finite element model to represent the inelastic behavior of the reinforcement. These relationships are shown in Fig. 10. No damage parameters were assigned to the reinforcement; only the elastic modulus (200 GPa) and Poisson’s ratio (0.3) were defined for the elastic region.

### Boundary, loading, analysis, and interactions conditions

In ABAQUS finite element analyses, the accurate definition of boundary conditions, loading protocols, and interaction properties is crucial for realistically replicating the experimental setup and capturing structural behavior. The first interaction defined was the bond between reinforcement and concrete, modeled using the embedded region constraint^46,52^. This ensured that the steel reinforcement remained fully integrated with the surrounding concrete throughout loading, constraining its degrees of freedom to those of the concrete and thereby providing realistic bond–slip behavior under monotonic loading^59^. At the loading points, rigid steel plates and loading bars were modeled and connected to the beam surfaces using a tie constraint, enabling the contacting surfaces to move together without relative slip. Similarly, reference points were assigned to the support locations and connected to the corresponding beam surfaces through coupling constraints, allowing for the extraction of reaction forces during the analysis. Surface-to-surface contact behavior was defined with hard contact in the normal direction to prevent penetration and a penalty friction coefficient of 0.35 in the tangential direction to represent steel–concrete interface friction.

Boundary conditions were defined to accurately replicate the experimental support conditions. Both ends of the beam were modeled as fully fixed (encastre), with all translational and rotational degrees of freedom restrained. No longitudinal translation was allowed at either support. In addition, the regions of the beam embedded within the support zones were also assigned fully constrained boundary conditions, preventing any displacement or rotation. This modeling approach ensured a consistent representation of the experimental setup and enabled an accurate simulation of fixed-end structural behavior.

**Fig. 11:**
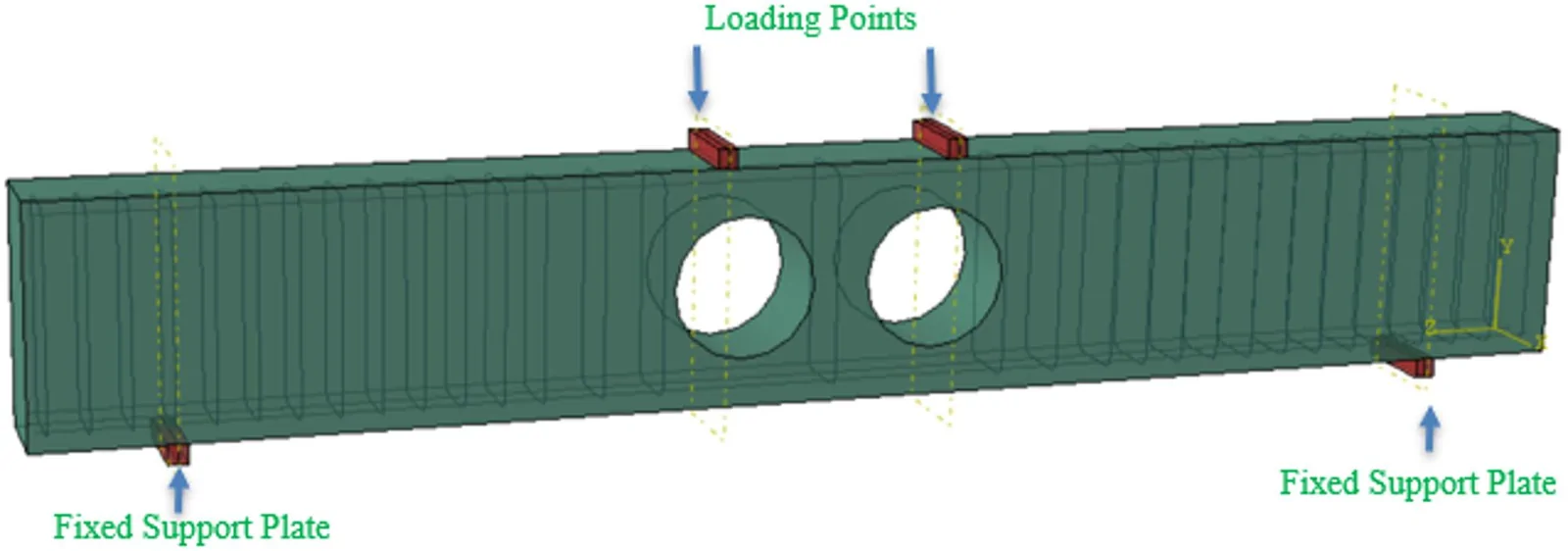
Views of the numerical modeling of N-BWO-360 sample.

The numerical loading setup was defined to replicate the experimental program, with two-point loading applied through rigid plates connected to reference points. Displacement-controlled loading was implemented in accordance with the FEMA 461 loading protocol^67^. The Dynamic Explicit procedure was adopted as the solution method. This approach is based on an explicit time integration scheme and is particularly suitable for highly nonlinear problems involving crack initiation, crack propagation, stiffness degradation, and damage evolution in reinforced concrete. It provides a robust numerical framework capable of capturing advanced nonlinear behavior while avoiding convergence difficulties commonly encountered in implicit solution methods. The selected analysis procedure, together with the defined contact and material modeling strategies, enabled a realistic simulation of load transfer, crack development, and progressive failure mechanisms consistent with experimental observations. Views of the numerical modeling of the samples are presented in Fig. 11.

### Meshing of numerical samples

**Fig. 12:**
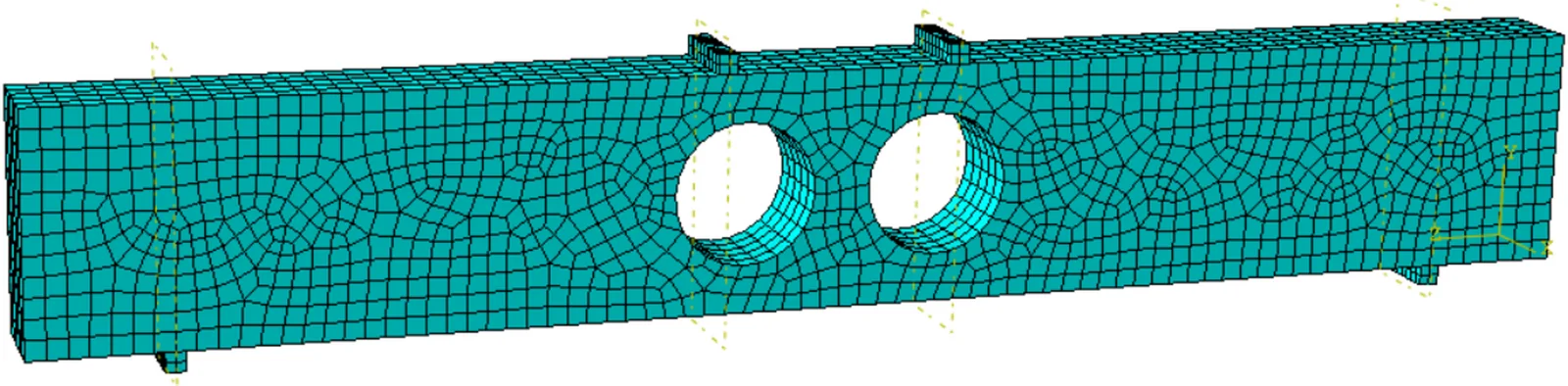
Mesh views of the numerical modeling of N-BWO-360 sample.

In the finite element modeling stage, mesh assignments and element type definitions were made for all material components to accurately represent the structural behavior. All materials were defined in three dimensions, and the reinforced concrete beam was modeled using C3D8R elements eight-node linear brick elements with three translational degrees of freedom per node and a single reduced integration point. The beams were discretized using cubic finite elements, while the reinforcement bars were modeled with T3D2 elements, which are 3D truss elements consisting of two nodes and one translational degree of freedom. To ensure consistency between concrete and reinforcement interaction, the mesh size was assigned as 50 mm for the concrete elements and 25 mm for the reinforcement bars based on the mesh sensitivity study results. This mesh configuration allowed accurate stress transfer within the embedded region constraint while maintaining computational efficiency. The loading and support plates were also modeled using C3D8R elements with mesh sizes compatible with the surrounding concrete discretization, ensuring compatibility with the beam geometry and the experimental bond lengths. A mesh sensitivity study was performed to determine the optimal mesh size for the concrete region. Following recommendations from the literature, cubic elements with an aspect ratio of 1:1 were tested at mesh sizes of 20, 25, 30, 40, and 50 mm. By comparing numerical load–displacement curves with experimental data and evaluating convergence behavior, 50 mm was identified as the optimum mesh size for the concrete elements, while 25 mm was adopted for the reinforcement bars, providing a balance between computational efficiency and accuracy in capturing cracking, crushing, and localized deformation patterns. To better capture the stress concentrations around the circular openings, local mesh refinement was applied. The mesh density was gradually increased near the hole edges, with smooth transitions to the global mesh to avoid abrupt stiffness changes. This refinement ensured accurate modeling of the critical regions where cracking and strain localization were expected. The adopted meshing strategy also ensured that the effective bond length between reinforcement and surrounding concrete in the numerical model matched that of the experimental specimens. The final finite element model and mesh configuration are presented in Fig. 12 using the N-BWO-360 specimen as a representative example, clearly illustrating the adopted mesh arrangement and discretization strategy in the vicinity of the circular opening.

#### Mesh sensitivity

**Fig. 13:**
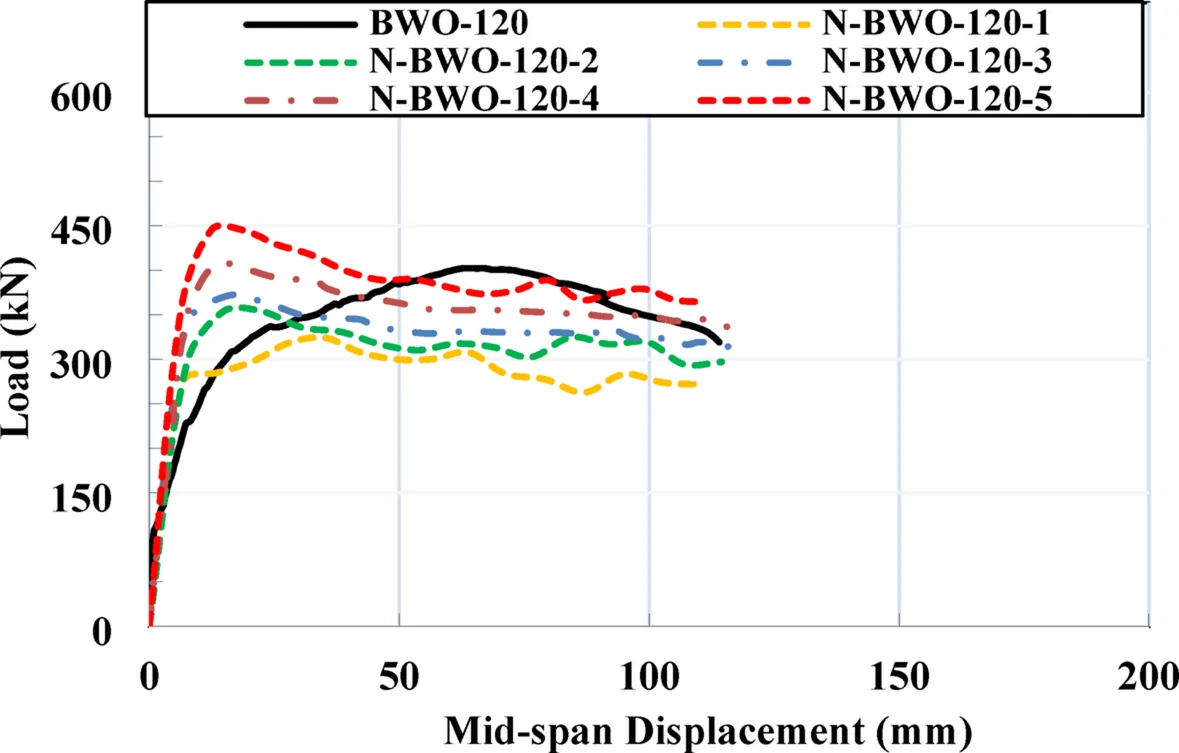
Load-displacement curves of analyses of sample BWO-120 at different mesh sizes.

Element types are defined for all materials in mesh assignments. All materials were assigned to 3D and the beam was modeled as C3D8R. C3D8R refers to modeling with 8 nodes and 1 reduced integration point with 3 displacement degrees of freedom. The beam is solved as a cubic finite element. Loading and suppor plates are resolved with the same properties as the beam, but with a higher mesh size. The reinforcements were analyzed as T3D2, that is, a 2-node 3D rod finite element with a single degree of freedom. The finite element analysis of the BWO-120 sample was considered as an example within the scope of the mesh sensitivity section. Analyses were made by changing the mesh sizes of the relevant sample as fine, medium and coarse. The mesh sizes of the relevant samples are presented in the table. Various mesh sizes were employed in the analysis for both rebar and concrete. The mesh sizes used are shown in Table 4. The load-displacement curves obtained according to the analysis results according to mesh sizes are given in Fig. 13.

**Table 4:** Mesh sizes of samples.

Sample Name	Rebar Mesh Size (mmxmm)	Concrete Mesh Size (mmxmm)
BWO-120-1	20 × 20	20 × 20
BWO-120-2	25 × 25	25 × 25
BWO-120-3	30 × 30	25 × 25
BWO-120-4	40 × 40	25 × 25
BWO-120-5	50 × 50	25 × 25

When the load-displacement curves are compared, decreasing the mesh size causes a decrease in the load value. For the BWO-120 sample, the 50 mm mesh size is the mesh size that is most compatible with the experimental results. As a result of the analyses, the 25 × 25 mm mesh size was determined in the reinforcement, while the 50 × 50 mm mesh size was determined in the sample concrete. These mesh sizes were also used in all other samples.

## Results

### Experimental results

This section presents and discusses the findings obtained from the experimental program conducted on reinforced concrete beam specimens with circular openings. First, the observed failure modes are illustrated, and the influence of the openings on crack propagation and damage mechanisms is evaluated. Subsequently, the load–displacement relationships derived from the tests are presented, followed by a comparative analysis between the experimental and numerical load–displacement curves. In addition, the moment–curvature relationships of the tested beams are examined to highlight variations in stiffness and post-yield behavior. The ductility capacities of the specimens are then calculated to compare their deformation abilities. Finally, the energy absorption capacities of the beams are determined, and the effects of openings on their energy dissipation characteristics are evaluated.

#### Failure modes of samples

**Fig. 14:**
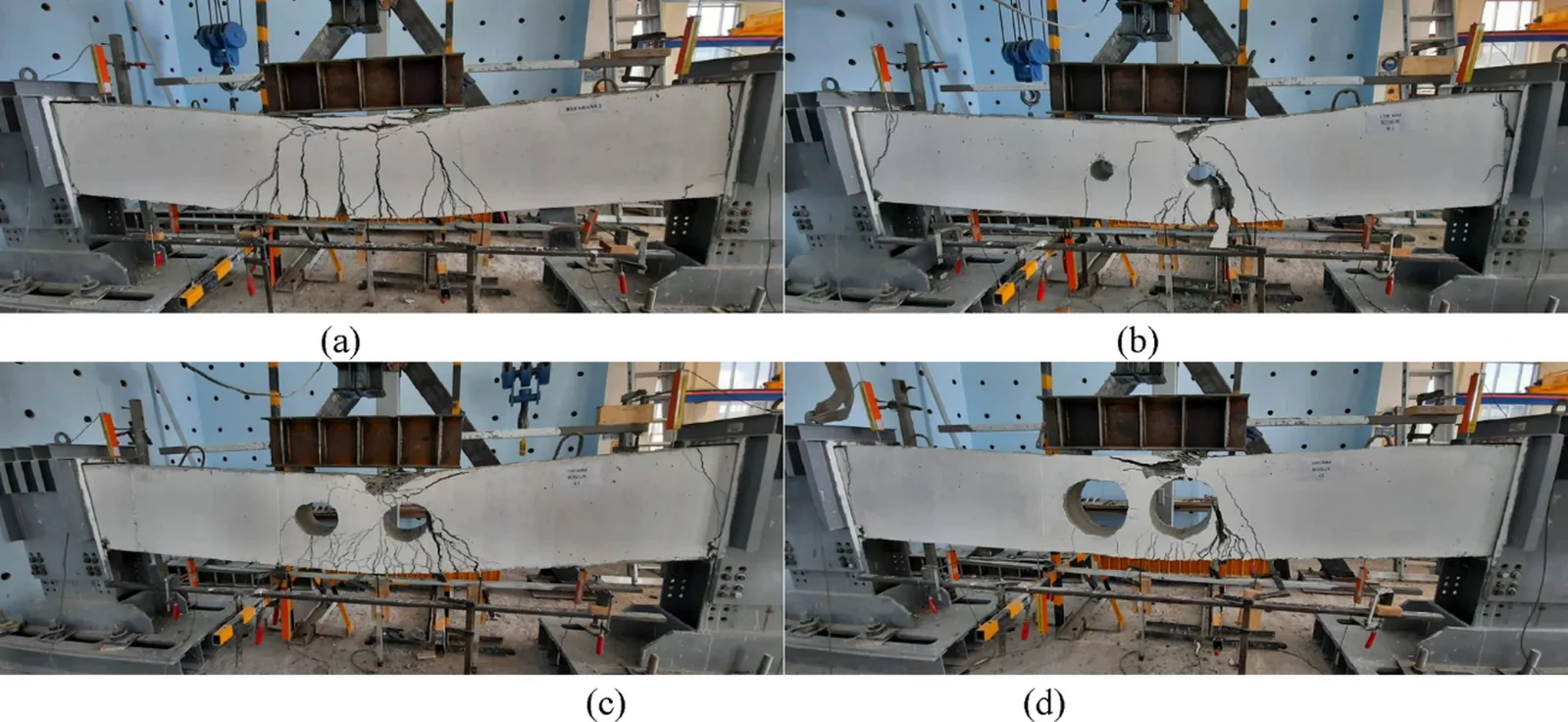
The failure mode in specimens; **a**) specimen “REF”; **b**) specimen “BWO-120”; **c**) specimen “BWO-240”; **d**) specimen “BWO-360”.

The experiments were conducted under monotonic four-point loading, using load control until the maximum load was reached, followed by displacement control after reinforcement yielding. Crack formation was monitored throughout each loading step, with newly developed cracks marked directly on the specimen surface for clarity. The tests continued until the beams reached their ultimate load capacity. After completion, all visible cracks were numbered on the specimen, and their widths were measured with a caliper. The corresponding load levels at which each shear or flexural crack formed were also recorded. The post-test failure modes of the reinforced concrete beams are presented in Fig. 14.

**Fig. 15:**
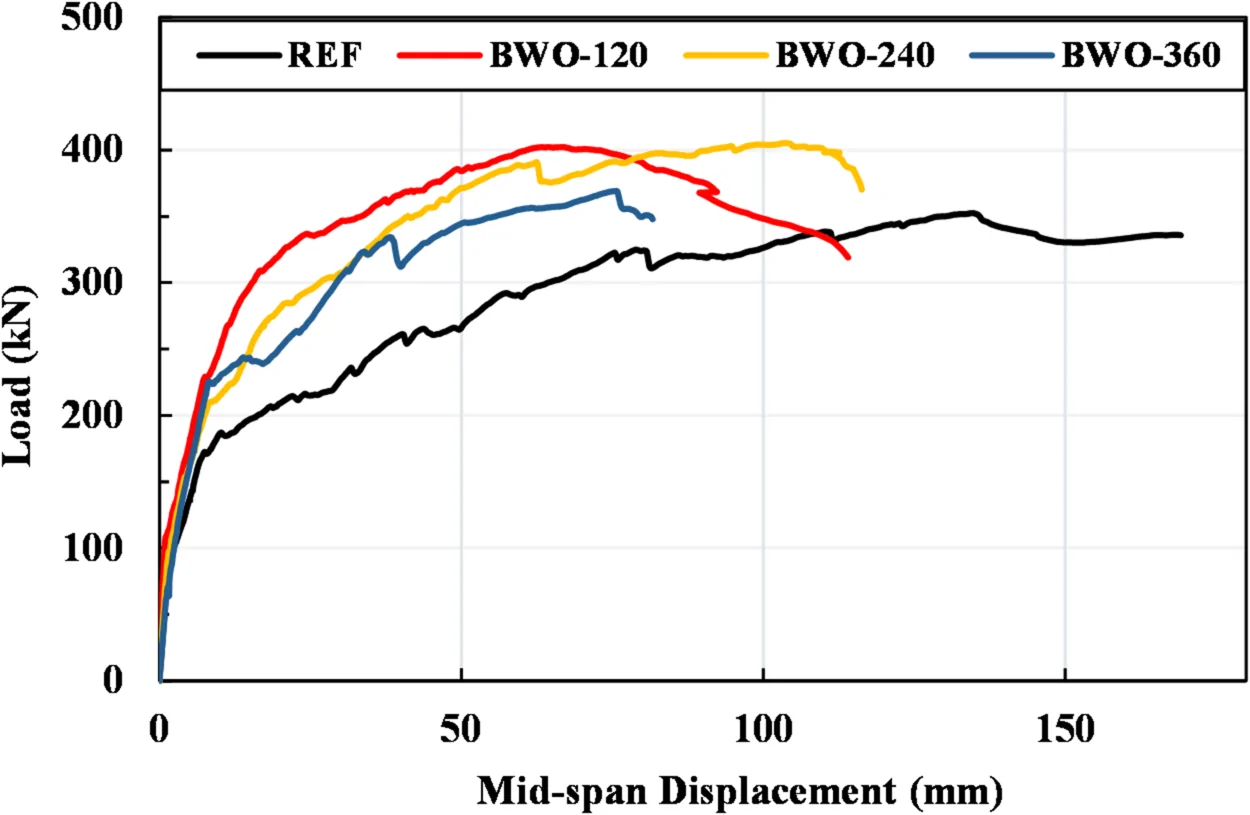
Load-midspan displacement curves of all specimens.

The failure mode observations revealed that all reinforced concrete beam specimens predominantly experienced flexural cracking initiated in the mid-span region, indicating bending-dominated behavior, except for specimen B5, which displayed a combined flexural–shear failure pattern. For the reference beams (R1 and R2), the first cracks occurred at relatively low displacements (3.39 mm and 2.05 mm, respectively), followed by stable crack propagation until yielding at 7.58 mm (R1) and 17.28 mm (R2). Their peak load capacities reached 390 kN and 352 kN, with corresponding maximum displacements of 54.3 mm and 134.7 mm, respectively. Beams with 20% height openings (B1 and B2) showed first crack loads around 133–135 kN, yielding at displacements between 7.11 mm and 12.16 mm. Failure occurred through the formation of well-defined flexural hinges within the pure bending region, with ultimate loads of 402–413 kN. Beams with 40% openings (B3 and B4) exhibited moderate stiffness reduction, with B3 sustaining significant deformation before collapse (103.7 mm at peak load) and B4 showing a slightly higher peak load of 405 kN but reduced post-yield ductility. The 60% opening beam (B5) reached a lower maximum load of 369 kN, with failure initiated by flexural cracks that quickly propagated into inclined shear cracks near the opening edges, indicating stress concentration and shear capacity degradation. Crack mapping showed that crack density and width increased proportionally with applied load, with cracks around openings being more localized and oriented toward load transfer paths. Load–displacement data confirmed that beams with larger openings experienced earlier stiffness degradation and reduced ductility compared to both reference and smaller-opening specimens. These experimental findings align with previous studies^6,12,13,24^, which reported that the introduction of openings accelerates crack initiation, increases stress concentration near discontinuities, and promotes combined flexural–shear failure mechanisms, particularly in members with higher opening ratios. These findings are consistent with Fig. 15, where localized damage zones and distinct crack propagation patterns can be clearly identified for each specimen.

#### Load-midspan displacement curves

Reinforced concrete beam specimens as a result of the experiment were compared by examining the load-midspan displacement curves. The curves of the specimens are given in Fig. 15 as a single graphic. The load value and fracture types of reinforced concrete beams are presented in Table 5.

**Table 5:** Load bearing capacities of reinforced concrete beams and fracture types.

Sample Name	First Crack Point		Yield Point		Maximum Load		Failure Mode			Type of Fracture
	Load (kN)	Disp. (mm)	Load (kN)	Disp. (mm)	Load (kN)	Disp. (mm)	Load (kN)	Disp. (mm)	Permanent Disp.(mm)	
REF	95	2.05	185	11.17	352	134.72	311	169.42	157.5	Flexural
BWO-120	135	2.77	224	7.11	402	63.48	318	114.23	127.57	Flexural
BWO-240	133	3.26	202	7.62	405	103.75	370	116.3	132.6	Flexural
BWO-360	130	3.48	224	8.00	369	75.6	350	81.41	83.22	Flexural-Shear

The experimental load–midspan displacement behavior exhibited consistent trends across all beam specimens, with notable differences depending on the opening size. For the reference (REF) beam without openings, initial cracking occurred at approximately 95 kN with a corresponding displacement of 2.05 mm, and the beam reached a maximum load of 352 kN. Beams with 120 mm and 240 mm openings (BWO-120 and BWO-240) demonstrated a load–displacement response broadly comparable to that of the reference specimen. The maximum loads of these beams were recorded as 402 kN and 405 kN, respectively, which are in a similar range to the reference beam. This indicates that openings up to approximately 40% of the beam depth do not lead to a pronounced reduction in load-carrying capacity. However, the displacement values at peak load were lower than those of the reference specimen, suggesting a reduction in deformation capacity despite the comparable strength levels.

In contrast, the beam with a 360 mm opening (BWO-360) exhibited a distinctly different structural response. Although its maximum load (369 kN) remained close to that of the reference specimen, the displacement at peak load was significantly reduced (75.6 mm compared to 134.7 mm for REF), indicating a pronounced loss in ductility. Furthermore, the failure mode transitioned from purely flexural to combined flexural–shear behavior, as also reflected in the fracture classification. The reduction in deformation capacity and permanent displacement (83.22 mm compared to 157.5 mm for REF) suggests a more brittle response and reduced energy absorption capacity.

**Fig. 16:**
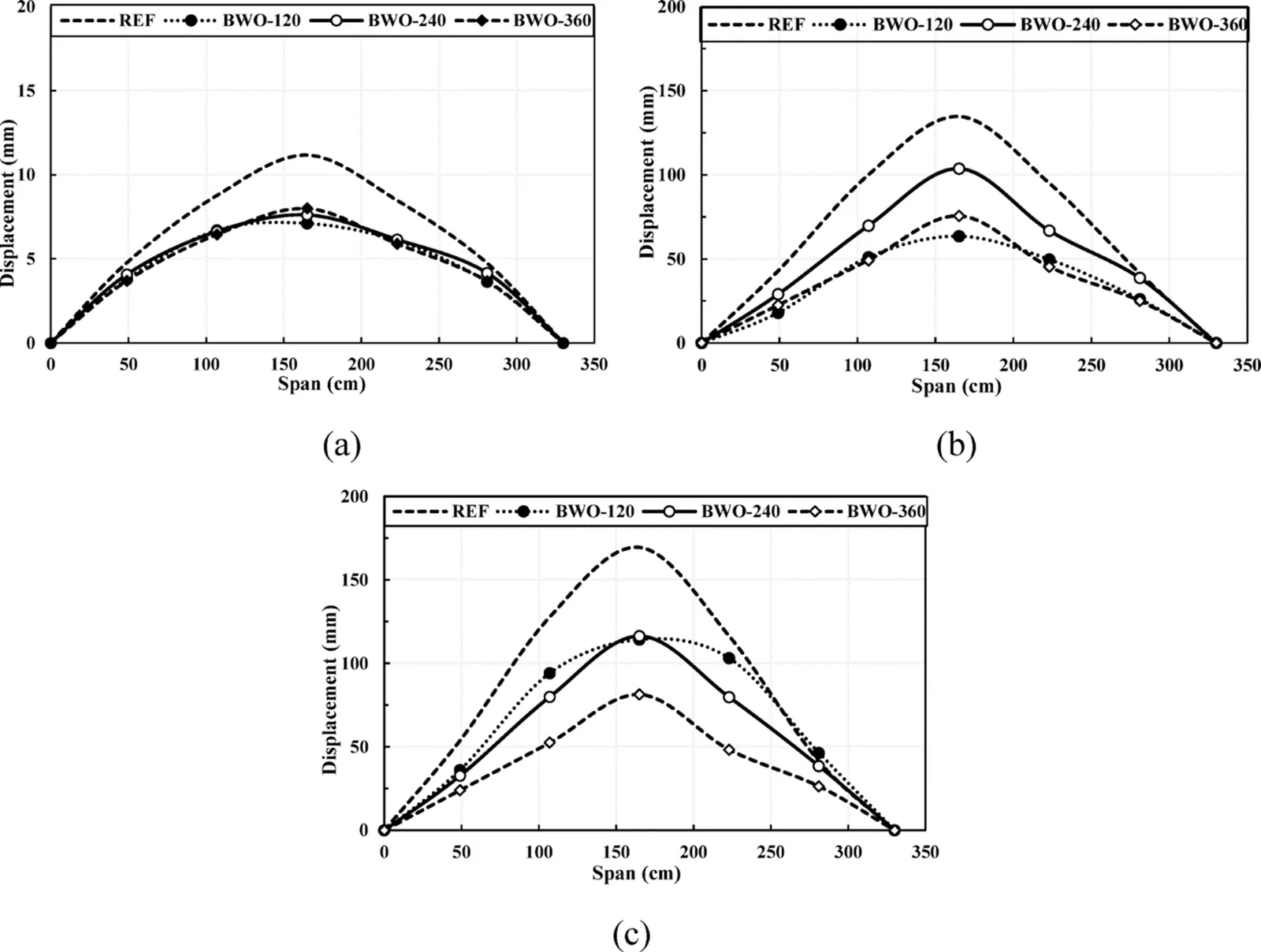
Distribution of deflections along the beam span at different loading stages: (**a**) yield load, (**b**) peak load, and (**c**) failure load.

Overall, the results indicate that while moderate opening sizes (up to ~ 40% of beam depth) do not significantly reduce load capacity, they still influence deformation behavior. Larger openings (≈ 60% of beam depth), however, lead to a marked reduction in ductility and a transition from flexural-dominated behavior to combined flexural–shear failure mechanisms. This transition reflects the increasing disruption of internal load transfer paths and the growing influence of stress concentrations around the opening. The deflection distributions along the beam span at yield, peak, and failure load levels are presented in Fig. 16.

The results clearly show that deflections near the support regions remain negligible for all specimens, confirming that the intended fixed-end boundary conditions were effectively achieved during the tests. The smooth and symmetric deflection shapes also indicate that the beams exhibit a consistent flexural response without significant local instabilities.

At the yield stage, all specimens demonstrate similar deflection patterns with relatively small differences, although the reference beam (REF) exhibits slightly higher deflection values compared to beams with openings. As the load increases to the peak level, the influence of opening size becomes more pronounced, with larger openings leading to reduced mid-span deflections and altered deformation distributions. This trend becomes even more evident at the failure stage, where the BWO-360 specimen exhibits significantly lower deflection compared to the other specimens. This behavior indicates a substantial reduction in deformation capacity due to the increased opening ratio, suggesting premature failure associated with section loss and reduced stiffness. Overall, the results confirm that opening size has a direct impact on the global deformation behavior, while the near-zero deflections at the supports verify the effectiveness of the fixed-end conditions.

#### Moment-curvature curves

**Fig. 17:**
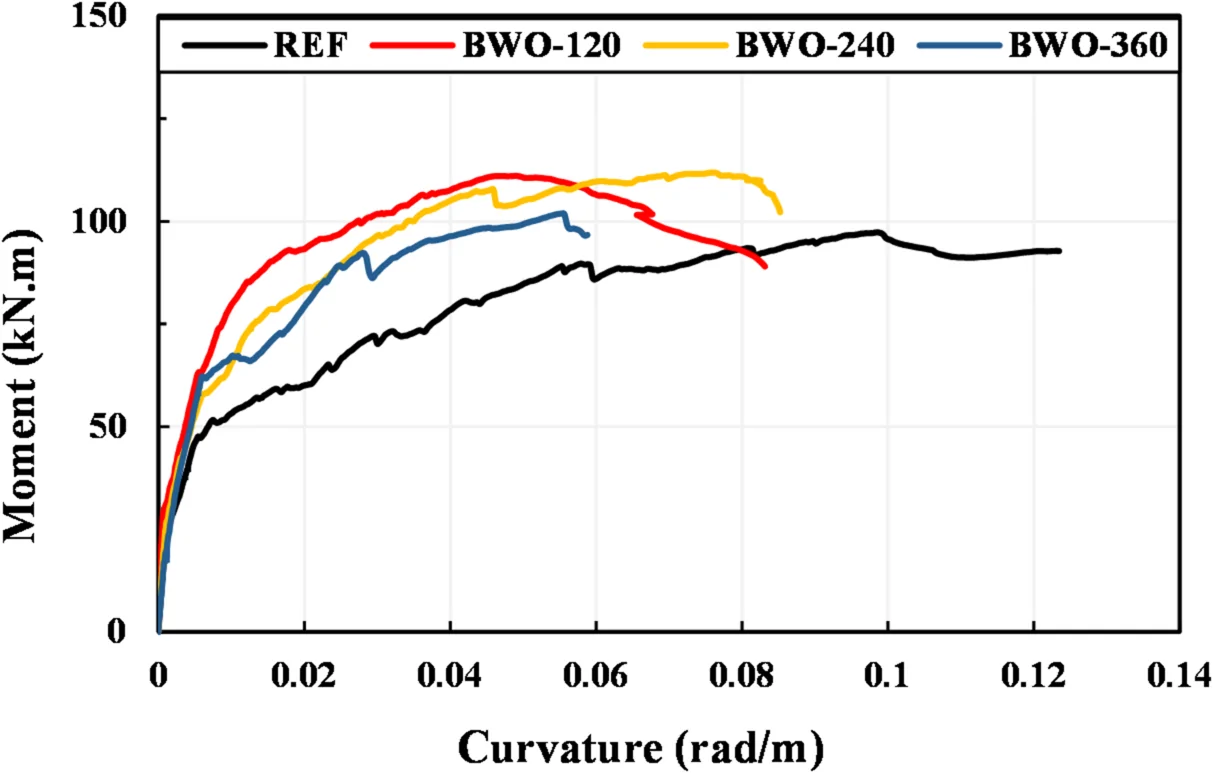
Moment-curvature curves of all specimens.

The moment-curvature curves of the reinforced concrete beam specimens are given in Fig. 17. The curvature values of reinforced concrete beams are calculated according to the moment cross-section effects and are given in Table 6. The moment–curvature relationship was used in this study to represent the flexural behavior of the beams by relating the applied bending moment to the curvature obtained from the measured displacement profile. The bending moment was calculated based on the four-point loading configuration, while the curvature was determined using a geometric approach by estimating the radius of curvature of the deformed beam axis^68–70^.This relationship provides a clear interpretation of stiffness degradation, crack development, deformation capacity, and curvature-based ductility behavior, and also serves as a consistent basis for comparison with the nonlinear finite element results^71^.

**Table 6:** Moment-curvature values of reinforced concrete beams.

Sample Name	Yield Point		Maximum Load		Ductility
	Moment (kNm)	Curvature (rad/m)	Moment (kNm)	Curvature (rad/m)	$\:\mu\:=\frac{{M}_{u}}{{M}_{y}}$
REF	51.1	0.00821	86.1	0.1238	15,10
BWO-120	62.3	0.00522	87.7	0.0837	16,03
BWO-240	55.8	0.00559	102.2	0.0852	15.24
BWO-360	61.8	0.00589	96.6	0.0597	10,14

The moment–curvature responses of the tested beams provide further insight into the influence of opening size on the flexural behavior and ductility characteristics. As presented in Table 6, the reference (REF) specimen exhibited a ductility ratio (µ) of 15.10, which reflects a stable and well-distributed deformation capacity. Beams with 120 mm and 240 mm openings (BWO-120 and BWO-240) showed ductility ratios of 16.03 and 15.24, respectively, which are comparable to that of the reference specimen. The variation in ductility remained within approximately ± 6% relative to the REF beam, indicating that openings up to approximately 40% of the beam depth do not significantly affect curvature-based ductility. This suggests that the flexural deformation capacity is largely preserved for moderate opening sizes. In contrast, the beam with a 360 mm opening (BWO-360) exhibited a substantial reduction in ductility, with a µ value of 10.14. This corresponds to a decrease of approximately 33% compared to the reference specimen. This significant drop indicates a clear transition in structural behavior, where the beam loses its ability to sustain large inelastic deformations. This reduction in ductility can be attributed to the disruption of the internal stress distribution and the reduction of the effective compression and tension zones due to the large opening. As the opening size increases beyond approximately 40% of the beam depth, the curvature capacity decreases and strain localization becomes more pronounced, leading to earlier failure and a more brittle response. Overall, the moment–curvature results confirm that while moderate openings (≤ 40% of beam depth) have a limited influence on ductility, larger openings (≈ 60%) result in a pronounced loss of deformation capacity and significantly reduce the structural resilience of the beams.

### Numerical results

**Fig. 18:**
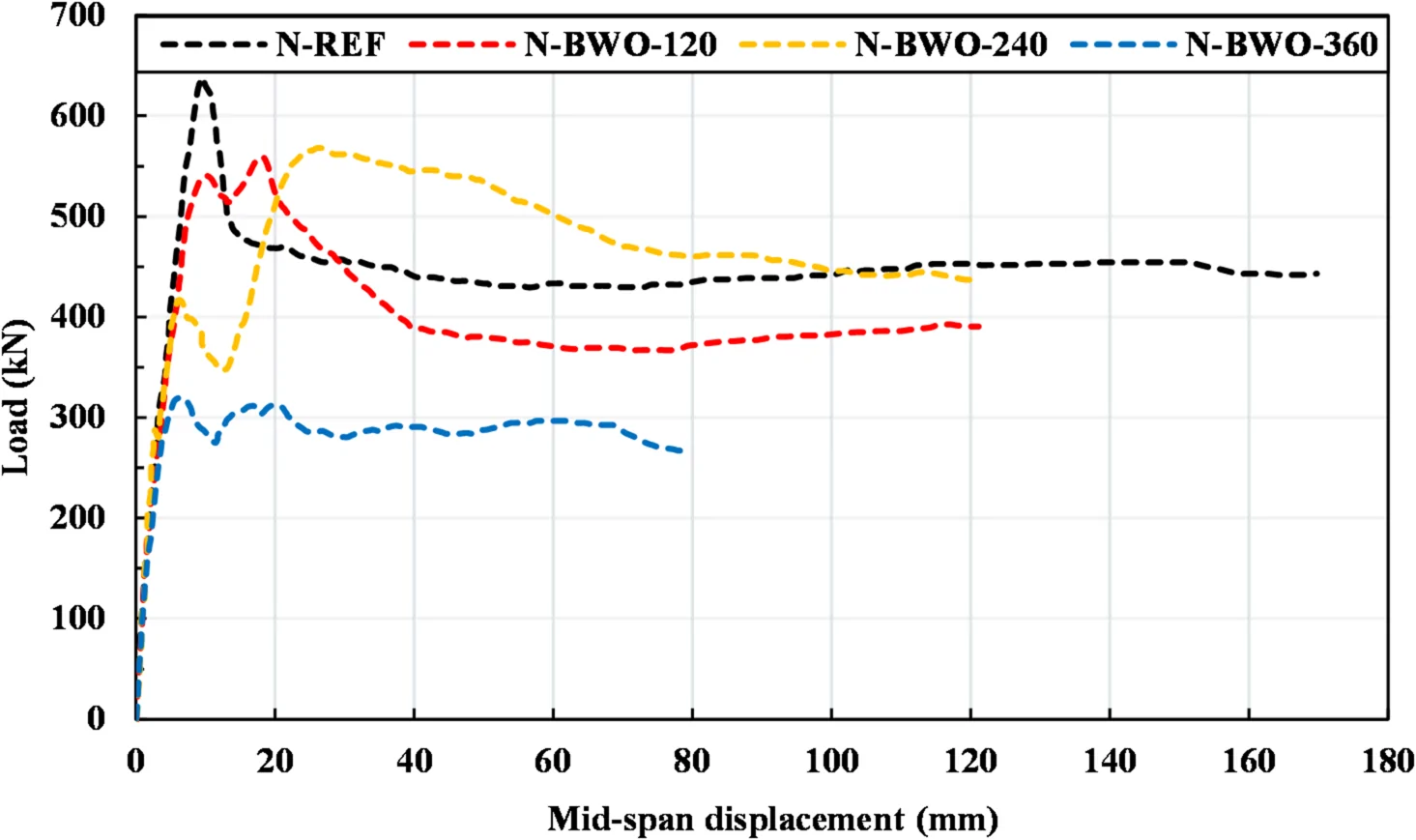
Load-displacement curves of numerical samples.

In this section, the finite element analyses of both the reference and opening specimens are presented to evaluate the structural response under flexural loading. The numerical simulations provide load–displacement curves that allow direct comparison with the experimental results and enable validation of the proposed modeling approach. Beyond the global behavior, the finite element model offers detailed insights into parameters that are challenging to monitor experimentally, such as damage progression in compression and tension regions, as well as the development of equivalent plastic strain under tension (PEEQT). These outputs enhance the understanding of local failure mechanisms and contribute to a more comprehensive assessment of the structural performance of beams with different opening ratios. The load-displacement curves of the numerical analysis results of reinforced concrete beam specimens are presented in Fig. 18.

**Fig. 19:**
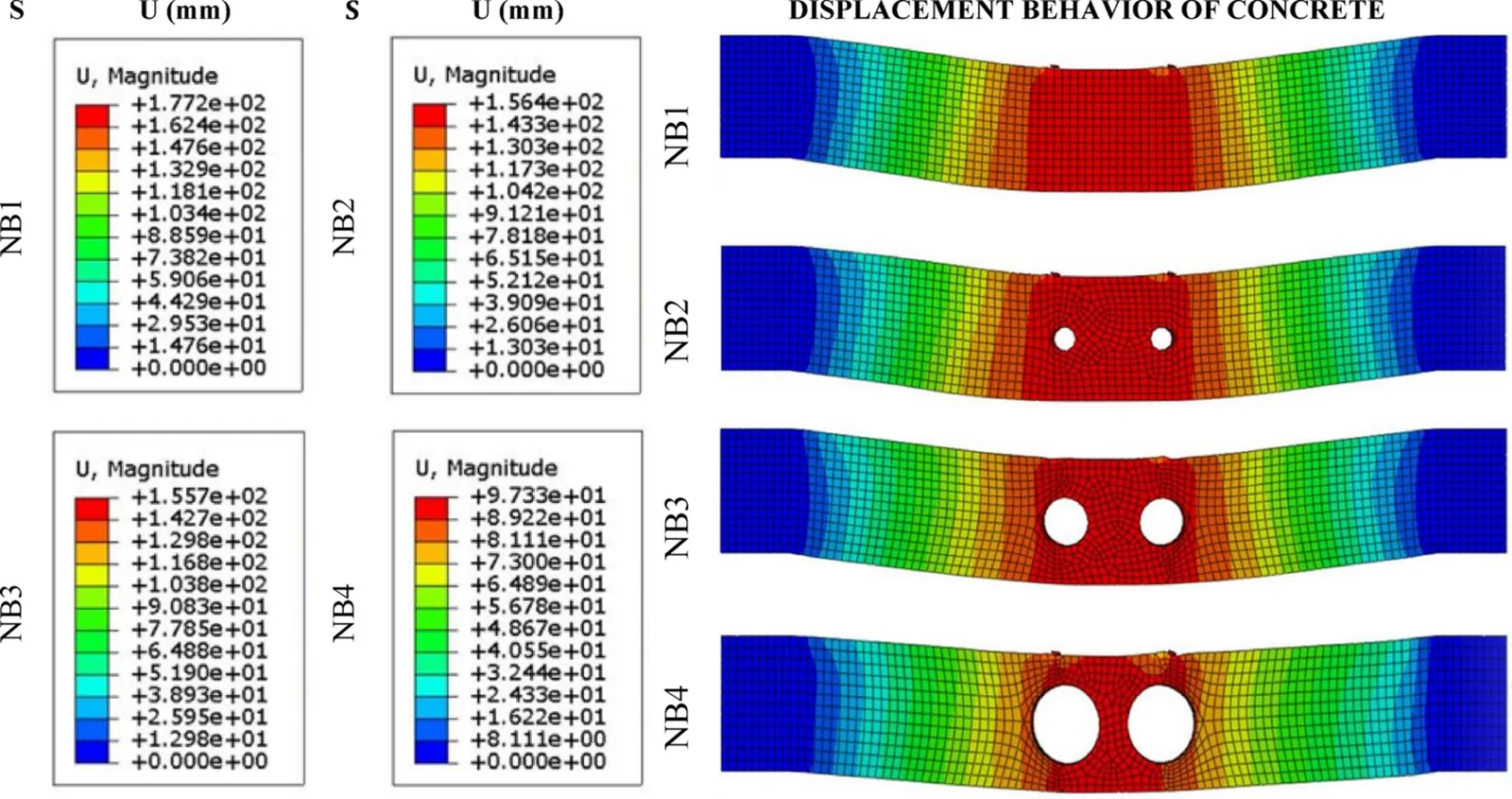
Displacement behavior of the numerical samples.

As illustrated in the load–displacement curves, all specimens exhibited comparable behavior within the elastic region. Among them, the reference specimen attained the highest capacity, reaching the yield point at 638 kN with a corresponding mid-span displacement of 9.55 mm. The specimen with a 20% opening yielded at 540 kN and 9.72 mm displacement, while the 40% opening specimen reached 416 kN with 6.42 mm displacement. The specimen with a 60% opening demonstrated the lowest performance, yielding at 320 kN with a displacement of 6.67 mm. Following the yield point, all specimens displayed a sudden drop in capacity, after which plastic deformation became dominant. This abrupt reduction in post-yield capacity is attributed to the influence of fixed-end boundary conditions. Overall, increasing the opening ratio resulted in progressive reductions in both yield load and displacement capacity. While the reference, 20% opening, and 40% opening specimens demonstrated relatively similar responses, the 60% opening specimen exhibited a significant reduction in both strength and ductility, highlighting the detrimental effect of larger openings on structural performance. Figure 19 presents the displacement behavior graphs of the numerical results of the beam specimens. As expected, no displacement occurred in the support regions due to the fixed boundary conditions defined at the ends, effectively simulating the fully restrained support behavior.

**Fig. 20:**
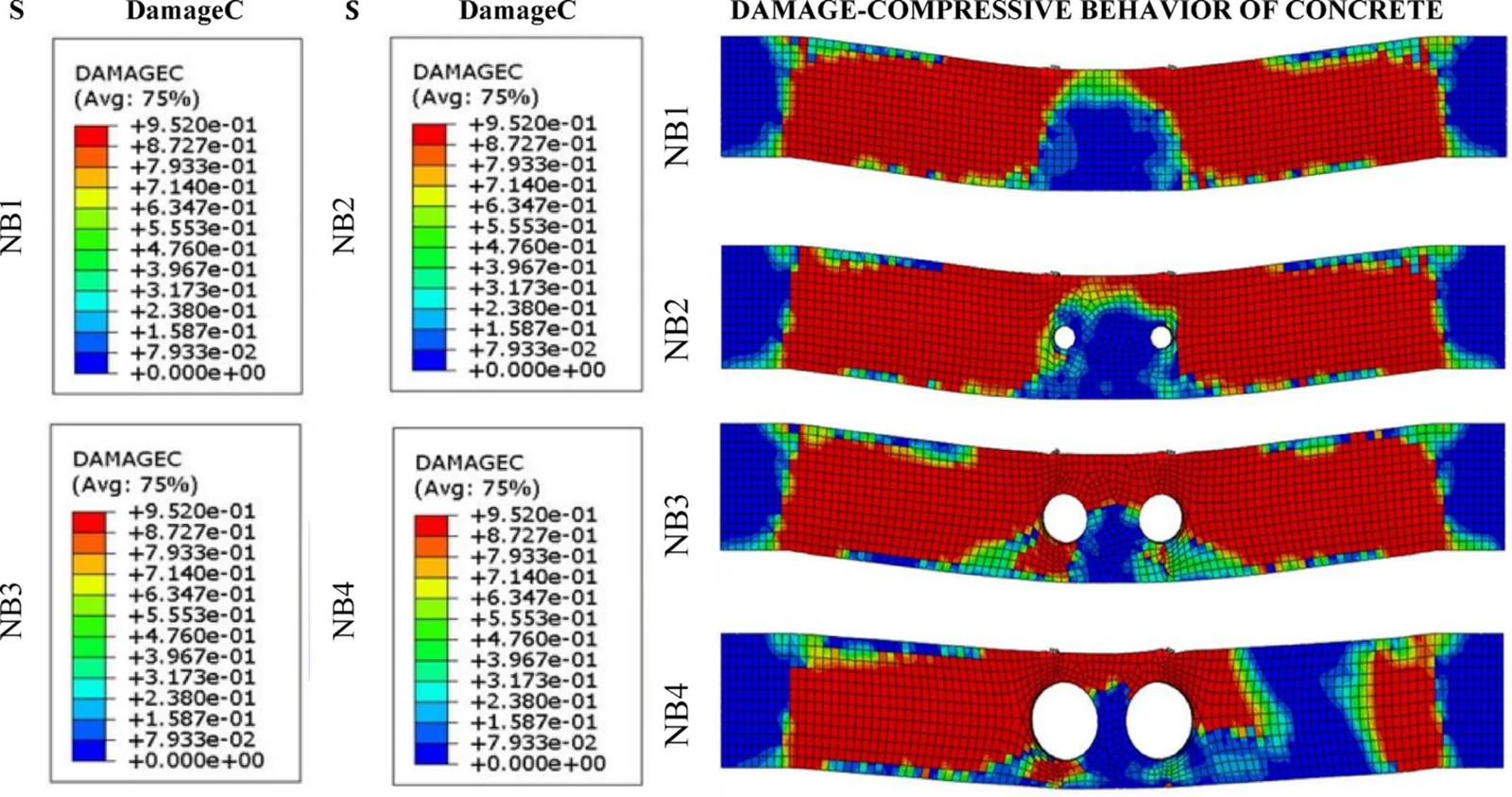
Damage-compressive behavior of the numerical samples.

The displacement values, expressed in millimeters, reveal that the maximum deformations are concentrated around the mid-span region where the openings are located, while the intensity of deformation decreases progressively towards the supports. Among the specimens, the reference beam (NB1) reached the highest displacement values, whereas beams with increasing opening ratios (NB2–NB4) exhibited lower displacement capacities. Particularly, NB4, with the highest opening ratio, displayed a significantly reduced mid-span displacement compared to the other beams, indicating the detrimental effect of larger openings on flexural deformation capacity. Overall, the comparison highlights that while the global displacement behavior remains similar across all models, the presence and size of openings substantially influence the magnitude of mid-span deflections. In Fig. 20, compressive-damage behavior graphs of the numerical results of the beam specimens are presented.

**Fig. 21:**
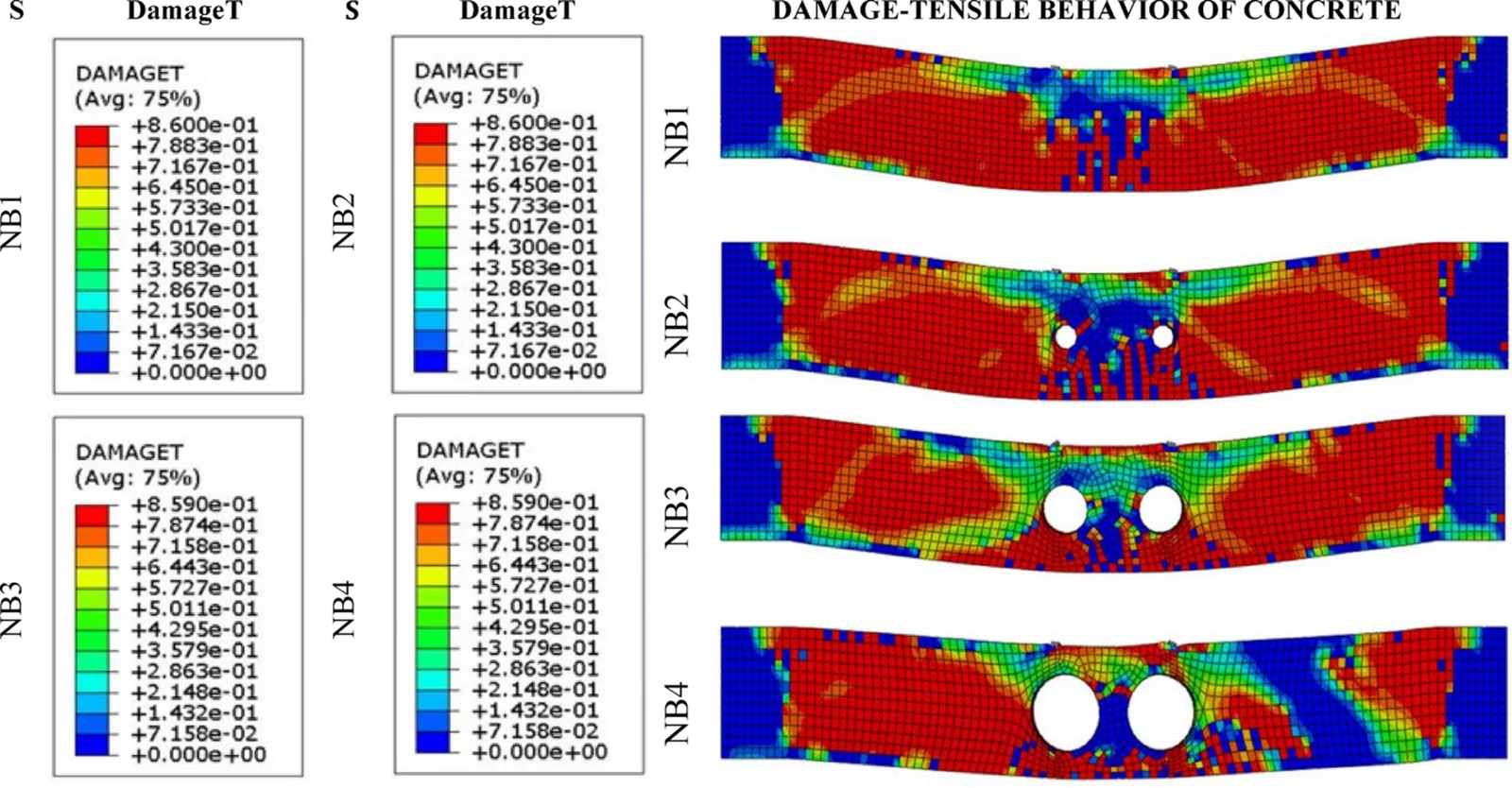
Damage-tensile behavior of the numerical samples.

In ABAQUS analyses, the Damage-T menu was used to evaluate tensile damage and crack development, and the Damage-C menu was used to determine compressive damage^72,73^. The compressive damage contours in Fig. 20 indicate that while the reference beam (NB1) exhibits relatively uniform stress distribution at the mid-span, beams with openings (NB2–NB4) demonstrate localized compressive damage around the holes. As the opening ratio increases, stress concentration becomes more severe, particularly in NB4, leading to premature crushing of the concrete. The support zones remained free from compressive damage due to fixed-end boundary conditions. Overall, the presence and size of openings significantly reduced the compressive resistance and accelerated damage propagation in the beams. In Fig. 21, tensile-damage behavior graphs of the numerical results of the beam specimens are presented.

**Fig. 22:**
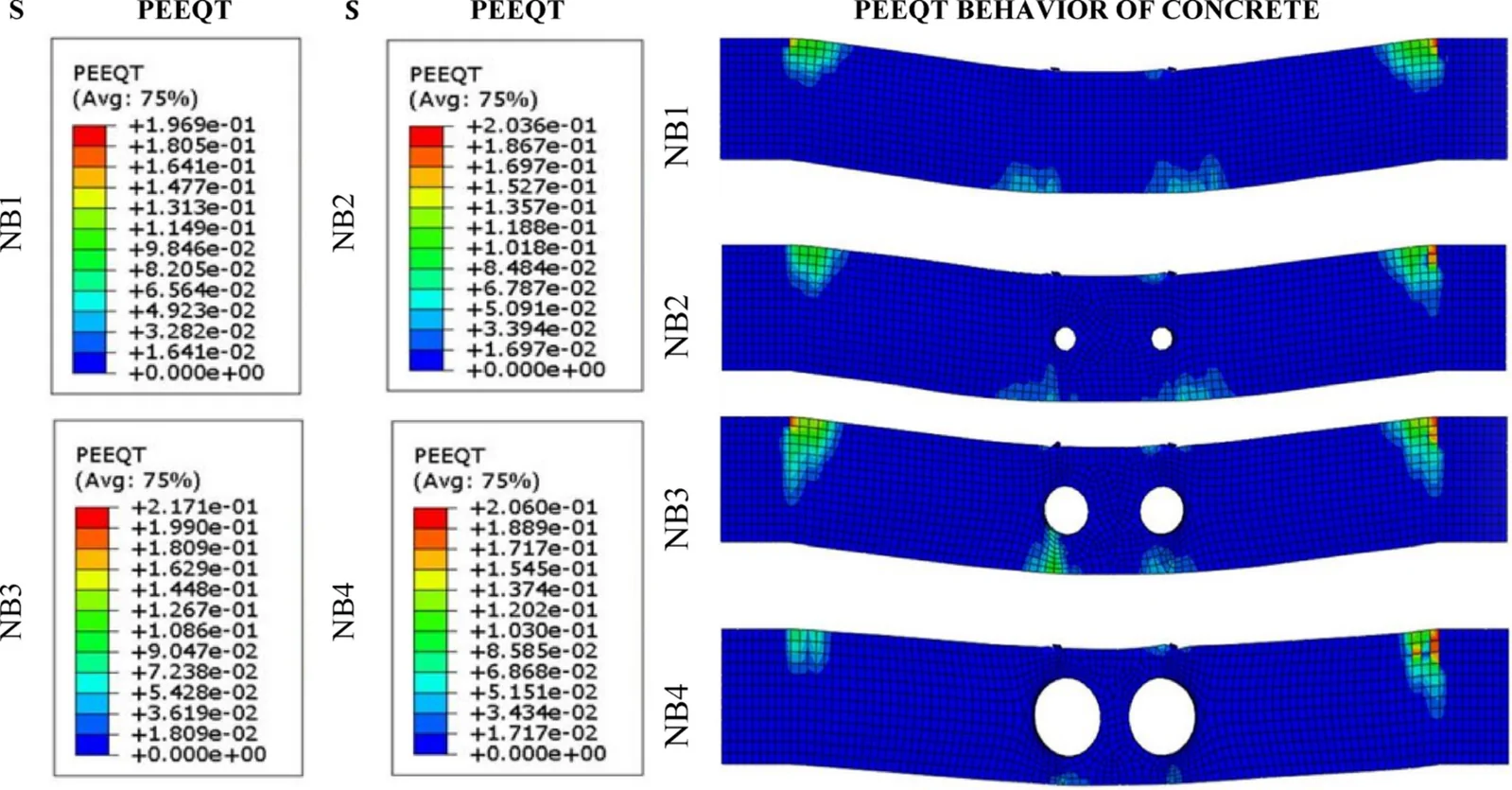
PEEQT behavior of the numerical samples.

Figure 21 presents the tensile damage behavior of the numerical specimens (NB1–NB4) based on the DAMAGET parameter in ABAQUS. In the reference specimen (NB1), crack propagation is primarily concentrated in the mid-span zone, governed by bending-induced tensile stresses. With the introduction of openings (NB2–NB4), a significant redistribution of stresses occurs, leading to localized tensile damage around the voids. These stress concentrations result in earlier crack initiation compared to the reference specimen. Particularly in NB3 and NB4, severe tensile damage zones are formed around and between the openings, which substantially disrupt the continuity of stress transfer along the beam section. The observed crack patterns indicate that as the opening ratio increases, the onset of tensile failure becomes more pronounced, and the overall damage severity intensifies. This outcome highlights the detrimental influence of voids on the structural integrity of beams, as they accelerate crack development and reduce the effective tensile resistance of the cross-section. Overall, the comparison clearly demonstrates that openings not only decrease the structural stiffness but also alter the failure mode by promoting localized tensile fractures. In Fig. 22, PEEQT behavior graphs of the numerical results of the beam specimens are presented.

**Fig. 23:**
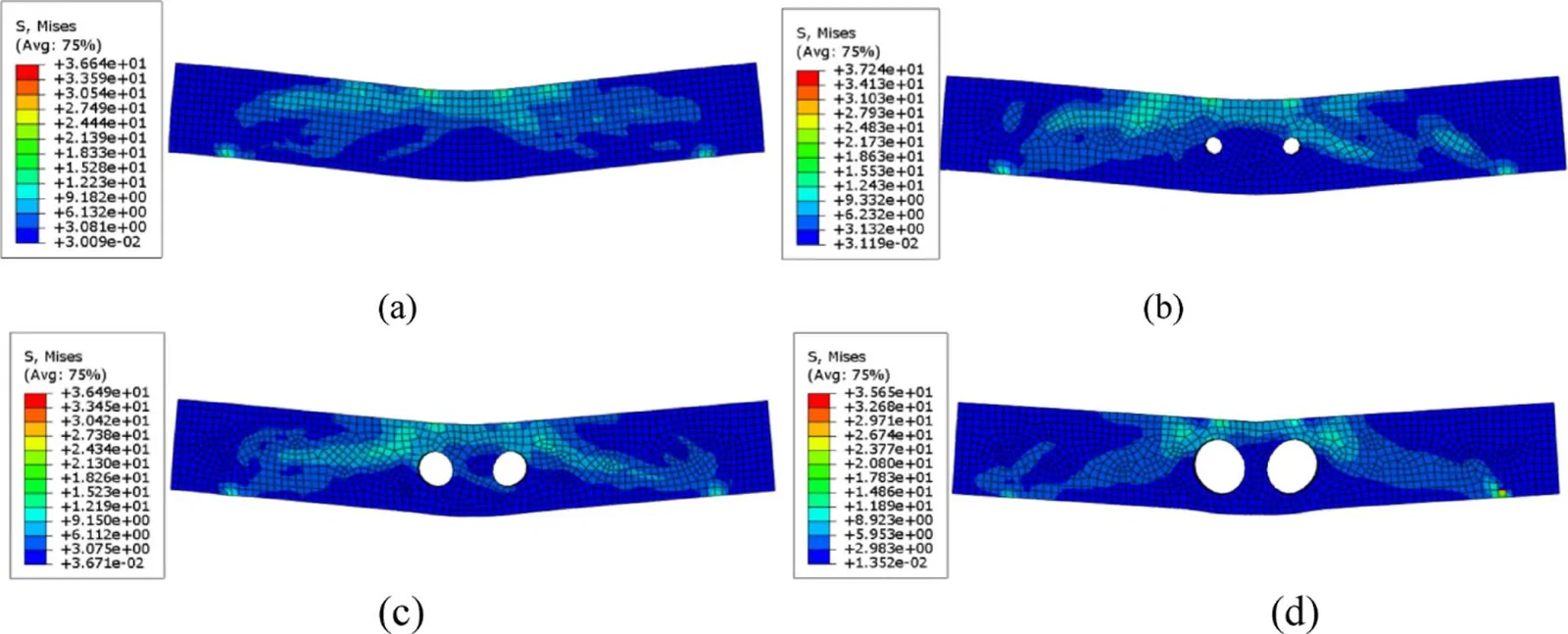
The Stress behavior mode in numerical specimens; (**a**) specimen “REF”; (**b**) specimen “BWO-120”; (**c**) specimen “BWO-240”; (**d**) specimen “BWO-360”.

The numerical evaluation of PEEQT (equivalent plastic strain in tension) revealed that plastic deformation zones are concentrated not only around the circular openings but also at the upper regions of the fixed supports. Interestingly, despite the reduction in displacement capacity with increasing opening ratio, the strain values did not show a corresponding decrease. This indicates that as the specimens approach their plastic hinge mechanism, the effect of openings reduces the overall displacement demand; however, the strain capacity tends to increase, leading to more localized plastic deformation. Such behavior suggests that the presence of openings accelerates the formation of critical damage regions by redistributing stresses and triggering higher strain accumulation around discontinuities. Concurrently, the restraint conditions at the fixed supports amplify strain localization, further confirming that both geometric discontinuities and boundary conditions govern the strain concentration mechanisms. These findings highlight that, although the global displacement demand is reduced, the local strain demand continues to rise, implying a higher degree of deformation and damage accumulation in the critical regions of the beams. Figure 23 shows the stress distributions of finite element specimens.

The numerical stress distributions obtained from the finite element analyses are compared with the experimentally observed crack patterns and failure modes in Fig. 23. For the REF specimen, the stress field is relatively continuous along the beam span, which is consistent with the flexural crack pattern observed experimentally. In BWO-120 and BWO-240, stress concentrations develop around the circular openings; however, the overall load transfer path remains largely continuous, explaining the flexural-dominated failure observed in the tests. For BWO-360, the stress distribution becomes more localized around the openings, indicating a significant disturbance of the internal load transfer mechanism. This agrees with the experimental observations, where the failure mode shifted from flexural behavior to combined flexural–shear behavior. These results show that increasing the opening size forces stress redistribution around the voids, reduces the effective load transfer region, and promotes localized damage development. Thus, the numerical stress contours are consistent with the experimentally observed crack patterns and provide a mechanical explanation for the reduced deformation capacity and more brittle response of specimens with larger openings.

### Comparison of experimental and numerical results

Comparison of the experimental and numerical results was performed on 4 specimens with similar characteristics. In the numerical study, a single analysis of the type samples was made and a total of 4 finite element models were created and analyzed. Load-displacement curves and energy consumption capacities were compared for experimental and numerical studies.

#### Experimental and numerical load-midspan displacement curves

**Fig. 24:**
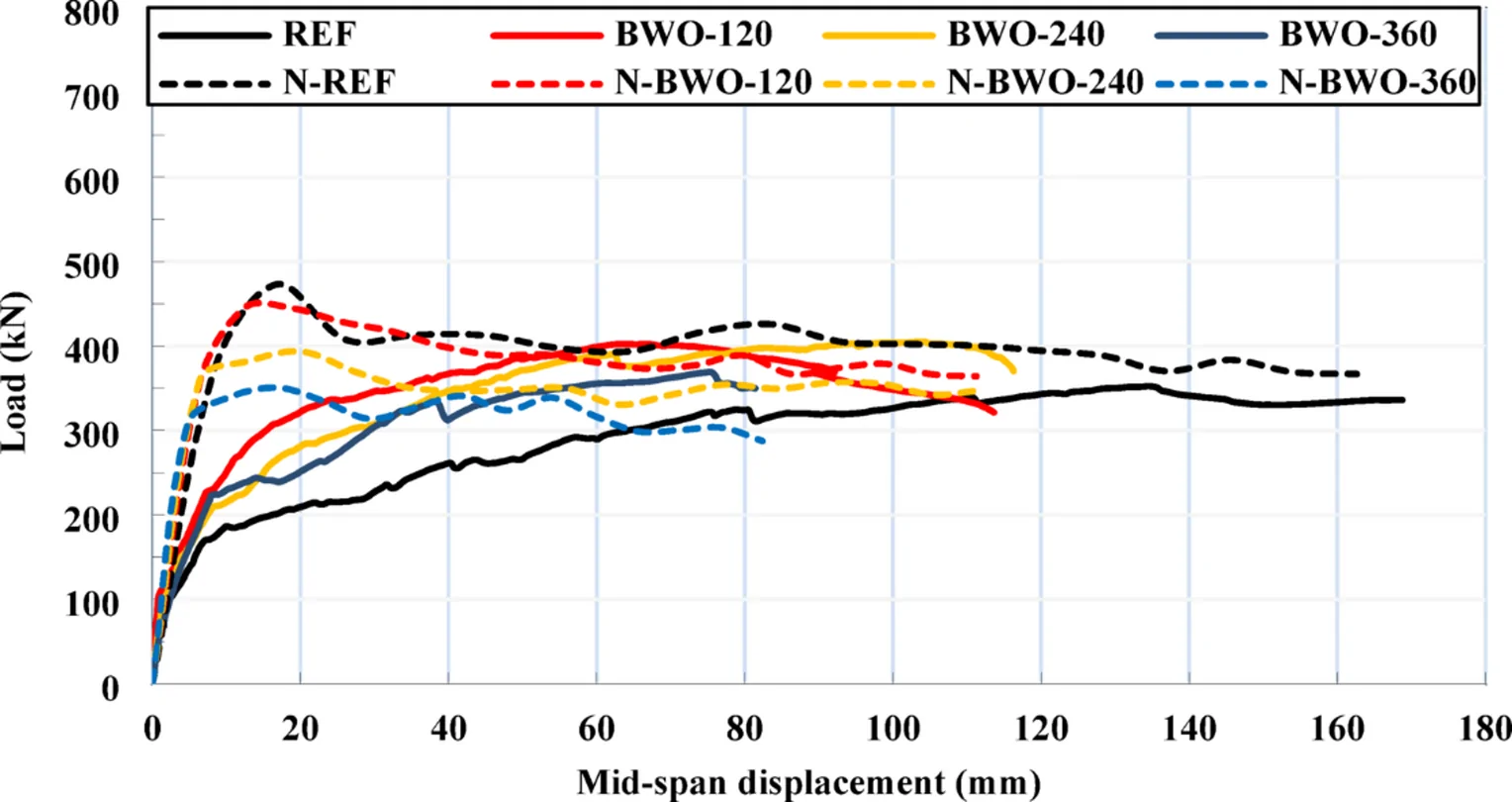
Load-displacement curves of experimental and numerical specimens.

As a result of the test and numerical analysis, the reinforced concrete beam specimens were compared by examining the load-midspan displacement curves. The curves of the specimens are given in Fig. 24 as a single graphic.

**Table 7:** Load bearing capacities of reinforced concrete beams and fracture types.

Sample name	First crack point		Yield point		Peak load		Failure mode	
	Load (kN)	Disp. (mm)	Load (kN)	Disp. (mm)	Load (Kn)	Disp. (mm)	Load (kN)	Disp. (mm)
REF	95	2.05	185	11.17	352	134.72	311	169.42
N-REF	93	2.08	371	8.33	473	16.74	367	162.64
BWO-120	135	2.77	224	7.11	402	63.48	318	114.23
N-BWO-120	118	2.11	355	6.32	444	19.12	364	111.29
BWO-240	133	3.26	202	7.62	405	103.75	370	116.30
N-BWO-240	120	2.13	360	6.40	393	20.18	348	112.07
BWO-360	130	3.48	224	8.00	369	75.6	350	81.41
NBWO-360	103	1.59	308	4.77	351	16.92	287	82.38

A quantitative comparison between the experimental and numerical results was performed using peak load, displacement at peak load, and post-peak/failure-stage response parameters. The experimental peak loads of REF, BWO-120, BWO-240, and BWO-360 were 352, 402, 405, and 369 kN, respectively, while the corresponding numerical values were 473, 444, 393, and 351 kN. Accordingly, the numerical-to-experimental peak load ratios were 1.34, 1.10, 0.97, and 0.95 for REF, BWO-120, BWO-240, and BWO-360, respectively. In percentage terms, the differences in peak load were + 34.4%, + 10.4%, − 3.0%, and − 4.9%, respectively.

The displacement values at peak load were also compared. The experimental peak-load displacements were 134.72, 63.48, 103.75, and 75.60 mm for REF, BWO-120, BWO-240, and BWO-360, respectively, whereas the corresponding numerical values were 16.74, 19.12, 20.18, and 16.92 mm. The numerical-to-experimental displacement ratios at peak load were therefore 0.12, 0.30, 0.19, and 0.22, respectively. These results indicate that the numerical model reproduces the load-capacity trend between specimens, while the displacement response at peak load remains more sensitive to modeling assumptions such as boundary idealization, local cracking, bond interaction, and post-cracking deformation localization.

For the post-peak/failure-stage response, the experimental failure loads were 311, 318, 370, and 350 kN, while the corresponding numerical values were 367, 364, 348, and 287 kN for REF, BWO-120, BWO-240, and BWO-360, respectively. The numerical-to-experimental ratios for failure load were 1.18, 1.14, 0.94, and 0.82. The corresponding failure-stage displacement ratios were 0.96, 0.97, 0.96, and 1.01, respectively. These values show that the numerical model captures the post-peak displacement response of all specimens with close agreement. Overall, the quantitative comparison indicates that the FE model reasonably represents the global load-carrying behavior, post-peak displacement response, and relative effect of opening size. The results also confirm that the transition from flexural behavior in REF, BWO-120, and BWO-240 to flexural–shear behavior in BWO-360 is consistently reflected in both the experimental observations and numerical simulations.

#### Experimental and numerical consumed energy amounts

A comparison of the experimental and numerical energy consumption results of the reinforced concrete beams reveals consistent trends with variation depending on the opening ratio. Experimentally, the reference specimen exhibited an absorbed energy of 49,272 kNmm, while the numerical result was calculated as 63,541 kNmm, corresponding to a numerical-to-experimental ratio of approximately 1.29. For the BWO-120 specimen, the experimental and numerical energy values were 41,475 kNmm and 42,345 kNmm, respectively, resulting in a ratio of 1.02. Similarly, for BWO-240, the experimental energy consumption was 43,235 kNmm, while the numerical value was 38,666 kNmm, corresponding to a ratio of 0.89. For the BWO-360 specimen, the experimental and numerical values were 24,364 kNmm and 25,044 kNmm, respectively, giving a ratio of 1.03.

In terms of relative changes, the experimental results indicate that the energy absorption capacity decreases from 49,272 kNmm in the reference specimen to 24,364 kNmm in the 60% opening specimen, corresponding to an approximate reduction of 50%. The numerical results show a similar trend, with energy decreasing from 63,541 kNmm to 25,044 kNmm, corresponding to a reduction of approximately 60%.

For the specimens with 120 mm and 240 mm openings, the energy values remain within a relatively close range to the reference specimen, indicating that moderate opening sizes do not significantly alter the overall energy dissipation behavior. In contrast, the BWO-360 specimen exhibits a notable reduction in energy consumption in both experimental and numerical results, indicating a clear change in structural response associated with larger openings.

**Fig. 25:**
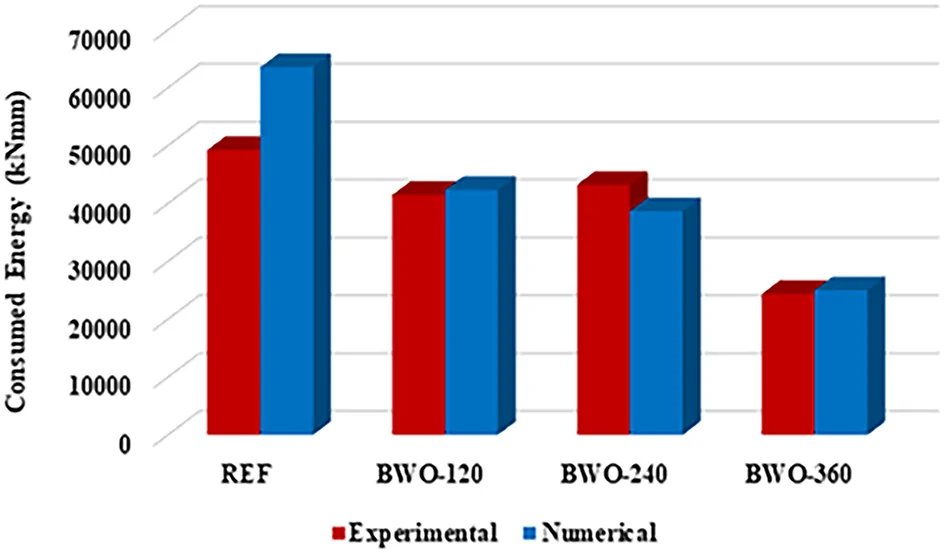
Energy consumption amounts of experimental and numerical specimens.

The comparison shows that both experimental and numerical results consistently capture the reduction in energy absorption capacity with increasing opening size. The relative behavior between specimens, particularly the transition observed in the 60% opening case, is represented similarly in both approaches. These findings confirm that the variation in energy consumption is strongly influenced by the increase in void ratio, with the most pronounced change occurring at higher opening levels. Energy consumption values are presented in Fig. 25.

## Conclusions

Openings are often introduced in reinforced concrete beams for functional purposes; however, they may compromise structural performance by creating stress concentrations and local weaknesses. In this study, the flexural response of beams with mid-span circular openings was investigated through both experimental testing and finite element simulations in ABAQUS. The following conclusions can be drawn:

- A comparison with major international design codes, including ACI 318, Eurocode 2 (EN 1992-1-1), CSA A23.3, and AASHTO LRFD, indicates that these standards do not prescribe explicit geometric limits for web openings in reinforced concrete beams, such as a maximum opening depth ratio or a minimum residual wall thickness. Instead, the influence of openings is addressed through reduced-section shear capacity, disruption of load transfer paths, and classification of the affected region as a discontinuity region (D-region). In such cases, design is typically performed using the strut-and-tie method (STM). Accordingly, the limits proposed in this study—namely restricting the opening size to approximately 40% of the beam depth and maintaining a minimum remaining wall thickness of approximately 20%—should be interpreted as experimentally and numerically derived practical design recommendations rather than direct code-prescribed limits. These thresholds represent transition boundaries beyond which conventional beam theory becomes inadequate and discontinuity-region behavior governs. Furthermore, openings located within approximately one member depth of supports or concentrated loads should be treated with particular caution and verified using STM-based design approaches.
- The increase in opening size significantly alters the internal load transfer mechanism of RC beams. Larger openings interrupt the natural stress flow, forcing compression and tension paths to deviate around the opening and leading to stress concentrations near its boundaries. This results in a reduction of the effective load-carrying cross-section and weakens the shear transfer mechanism, particularly by disrupting the formation of diagonal compression struts. Consequently, the structural behavior transitions from conventional beam action to a disturbed stress state, characterized by earlier cracking, stiffness degradation, and reduced load capacity.
- Opening size plays a critical role in governing the structural behavior of RC beams.
- Openings up to approximately 40% of the beam depth maintain load-carrying capacity at levels comparable to the reference beam, while larger openings significantly alter the structural response.
- Ductility and deformation capacity are more sensitive to opening size than load capacity. Although peak load values remain relatively close for moderate openings, displacement capacity, curvature ductility, and energy absorption decrease with increasing opening ratio.
- A clear transition in failure mechanism is observed beyond a critical opening ratio.
- Beams with openings exceeding approximately 40% of the beam depth exhibit a shift from flexural-dominated behavior to combined flexural–shear response, accompanied by localized damage and reduced deformation capacity.
- Wall thickness above the opening is a key parameter controlling structural integrity.
- Maintaining a minimum wall thickness of approximately 20% of the beam depth helps preserve the interaction between reinforcement and surrounding concrete and supports stable structural behavior.
- The numerical model provides a reliable representation of global structural behavior.
- The finite element analyses successfully capture load redistribution, stress concentration around openings, crack development, and failure mode transitions observed in the experiments.
- Energy absorption capacity decreases with increasing opening ratio.
- While specimens with moderate openings exhibit comparable energy dissipation behavior to the reference beam, specimens with large openings show a pronounced reduction in energy capacity, indicating reduced structural resilience.
- The adopted numerical framework is suitable for parametric investigations.
- The validated FE model enables efficient evaluation of opening-related parameters and can reduce the need for extensive experimental testing in future studies.
- From a design perspective, practical limits can be defined within the scope of this study. Openings in flexural regions should preferably be limited to approximately 40% of the beam depth, and a minimum wall thickness of about 20% should be maintained to ensure acceptable structural performance.

**Recommendations**:

- For practical engineering design, the results suggest limiting opening ratios to 40% of the beam depth, and ensuring adequate reinforcement detailing around openings.
- Finite element analysis calibrated with experimental data can be used as a robust tool for pre-design evaluation of opening effects, thus saving time and resources.
- Future studies should investigate the effects of cyclic and seismic loading, alternative reinforcement arrangements, and non-circular opening shapes to extend the applicability of the presented conclusions.

## Supplementary Information

Below is the link to the electronic supplementary material.

Supplementary Material 1

Supplementary Material 2

Supplementary Material 3

Supplementary Material 4

Supplementary Material 5

Supplementary Material 6

## Data Availability

The datasets generated and/or analyzed during the current study are available from the corresponding author on reasonable request.

## Abbreviations

OPC: Ordinary Portland Cement
FEM: Finite Element Method
RC: Reinforced Concrete
FEA: Finite Element Analysis
CFRP: Carbon-Fiber- Reinforced Polymer
SDEG: Scaler Stifness Degradation
C3D8R: Eight-node hexahedral elements
T3D2: A model 3D bar finite elements with two nodes and one degree of freedom
$\:{b}_{t}$: The ratio of plastic strain to inelastic strain
$\:{f}_{ct}$: Concrete tension strength
$$\:{w}_{o}$$: Crack width
$$\:\varDelta\:$$: Displacement
$$\:{d}_{c}$$: Damage parameter
$\:{f}_{b0}/{f}_{c0}$: The ratio of initial equibiaxial compressive yield stress to initial uniaxial compressive yield stress
K: $\:{K}_{c}$, the ratio of the second stress invariant on the tensile meridian
$$\:\varphi\:$$: Dilation angle
$$\:\upsilon\:$$: Viscosity parameter
$$\:\epsilon$$: Eccentricity
$\:{\epsilon\:}_{c}$: Strain of concrete
$\:{\epsilon\:}_{c}^{in}$: Inelastic strain of concrete
$\:{\sigma\:}_{c}$: Compression stress of concrete
$\:{E}_{0}$: Elastic modulus of concrete
$\:{d}_{c}$: Damage parameter of concrete
$\:{\epsilon\:}_{c}^{pl}$: Plastic strain of concrete
$\:{b}_{c}$: The ratio of plastic strain to inelastic strain
$$\:{\sigma\:}_{t}$$: Tension stress
