# Supplementary material for: Nonlinear behavior of fixed-end RC beams with circular openings based on experimental and finite element analysis
Source: Sci Rep. 2026 Jun 11;16:26608. doi: 10.1038/s41598-026-56645-9 (PMC13503886; doi:10.1038/s41598-026-56645-9)
Supplement: Supplementary file 1 [file 41598_2026_56645_MOESM5_ESM.pdf]

APC Waiver Request

Dear Editorial Office,

I hope this message finds you well.

I am writing to respectfully request a full waiver of the Article Processing Charge (APC) for our manuscript submitted to *Scientific Reports*. We highly value the opportunity to publish our work in such a respected journal; however, it is unfortunately not financially possible for us to cover the APC.

We are researchers based in Türkiye, where the economic conditions in recent years have been severely affected by extremely high inflation rates and significant currency depreciation. As a result, the purchasing power of academic salaries has dramatically declined. Under the current circumstances, the APC required for publication represents an amount equivalent to several months of a researcher's salary.

In addition, our institution currently does not provide financial support for open-access publication fees, and we do not have any active research grants or project funds that can be used to cover APC costs. Despite our continuous efforts to obtain external funding, financial support specifically allocated for publication charges is unfortunately unavailable.

It is important to emphasize that the inability to pay the APC is not due to a lack of willingness to support open-access publishing, but rather due to genuine financial limitations and the absence of institutional or project-based funding. Therefore, covering the APC through personal financial resources is not feasible.

Publishing our research in *Scientific Reports* would allow our findings to reach a broad international scientific audience and contribute to the dissemination of knowledge in our field. However, without a full APC waiver, it would unfortunately be impossible for us to proceed with publication.

For these reasons, we respectfully request the editorial office to consider granting us a full waiver of the APC, taking into account the economic challenges and the lack of available funding to support publication costs.

We sincerely appreciate your time and consideration, and we would be very grateful for your support.

Kind regards,

Arş. Gör. Hüseyin Hilmi ASLANBAY
ERÜ Mühendislik Fakültesi
İnşaat Mühendisliği Bölümü
Yapı Anabilim Dalı

Corresponding Author
Huseyin Hilmi Aslanbay
Research Assistant
Department of Civil Engineering
Erciyes University
Kayseri, Türkiye

Scanned with CamScanner
