# Supplementary material for: Nonlinear behavior of fixed-end RC beams with circular openings based on experimental and finite element analysis
Source: Sci Rep. 2026 Jun 11;16:26608. doi: 10.1038/s41598-026-56645-9 (PMC13503886; doi:10.1038/s41598-026-56645-9)
Supplement: Supplementary file 2 [file 41598_2026_56645_MOESM6_ESM.pdf]

APC Waiver Request

Dear Editorial Office,

To whom it may concern,

The manuscript titled *"Experimental and Numerical Analysis of the Effect of Mid-Span Circular Openings on Behavior in RC Beams with Fixed Support"* prepared by Research Assistant Huseyin Hilmi Aslanbay was conducted within the scope of a research project supported by the Scientific Research Projects Coordination Unit (BAP) of Erciyes University.

The project was led by Prof. Dr. Fatih Altun as the principal investigator, while Huseyin Hilmi Aslanbay participated in the project as a researcher. The study is also based on the author's master's thesis research.

The financial support provided by the BAP project was used in 2021 for the production of experimental specimens and the establishment of the experimental setup required for the study. However, the project budget did not include any allocation for Article Processing Charges (APC) required for Open Access publication.

Furthermore, due to the limited project budget, some of the expenses related to the experimental work were partially covered personally by the researchers.

Therefore, there is currently no available project or institutional funding to cover the Open Access publication fee associated with this manuscript.

In light of these circumstances, we kindly request Springer Nature to consider granting a full waiver of the Article Processing Charge (APC) for this manuscript.

Sincerely,

Professor Zulkuf KAYA

Head of Department
Department of Civil Engineering
Erciyes University
Kayseri, Türkiye

Prof.Dr.Zülküf KAYA
ERÜ İnşaat Mühendisliği
Geoteknik Anabilim Dalı

Scanned with CamScanner
