# Supplementary material for: Nonlinear behavior of fixed-end RC beams with circular openings based on experimental and finite element analysis
Source: Sci Rep. 2026 Jun 11;16:26608. doi: 10.1038/s41598-026-56645-9 (PMC13503886; doi:10.1038/s41598-026-56645-9)
Supplement: Supplementary file 3 [file 41598_2026_56645_MOESM2_ESM.docx]

**Highlights**

- Experimental and numerical evaluation of RC beams with circular openings
- Increasing opening ratio significantly reduces ductility and energy capacity
- ABAQUS simulations validated with full-scale experimental beam results
- Critical degradation observed for openings exceeding 40% of beam height
- Fixed support boundary conditions provided realistic structural representation
