# Supplementary material for: Nonlinear behavior of fixed-end RC beams with circular openings based on experimental and finite element analysis
Source: Sci Rep. 2026 Jun 11;16:26608. doi: 10.1038/s41598-026-56645-9 (PMC13503886; doi:10.1038/s41598-026-56645-9)
Supplement: Supplementary file 5 [file 41598_2026_56645_MOESM4_ESM.docx]

**Title:**

Fixed-End Nonlinear Response of RC Beams with Circular Openings: Experimental Verification and FE-Based Prediction

**Corresponding Author:**

Huseyin Hilmi Aslanbay

Research Asst.

Erciyes University, Department of Civil Engineering, Kayseri, Turkey

[aslanbay@erciyes.edu.tr](mailto:aslanbay@erciyes.edu.tr)

ORC ID: 0000-0001-9739-4699

**Authors:**

Huseyin Hilmi Aslanbay, Fatih Altun

**Declaration of Competing Interest**

The authors declare that they have no known competing financial interests or personal relationships that could have appeared to influence the work reported in this paper.

**Data availability**

The datasets generated and/or analyzed during the current study are available from the corresponding author on reasonable request.

**Acknowledgement / Funding**

This study was supported by Erciyes University Scientific Research Projects with project code FYL-2020-10446.
